# Interferon-gamma inhibits influenza A virus cellular attachment by reducing sialic acid cluster size

**DOI:** 10.1016/j.isci.2022.104037

**Published:** 2022-03-06

**Authors:** Carol Ho-Yan Fong, Lu Lu, Lin-Lei Chen, Man-Lung Yeung, Anna Jinxia Zhang, Hanjun Zhao, Kwok-Yung Yuen, Kelvin Kai-Wang To

**Affiliations:** 1 State Key Laboratory for Emerging Infectious Diseases, Carol Yu Centre for Infection, Department of Microbiology, Li Ka Shing Faculty of Medicine, The University of Hong Kong, Pokfulam, Hong Kong Special Administrative Region, People’s Republic of China; 2 Department of Microbiology, Queen Mary Hospital, Pokfulam, Hong Kong Island, People’s Republic of China

**Keywords:** Biological sciences, Immunology, Microbiology

## Abstract

The mucosal antiviral role of type I and III interferon in influenza virus infection is well established. However, much less is known about the antiviral mechanism of type II interferon (interferon-gamma). Here, we revealed an antiviral mechanism of interferon-gamma by inhibiting influenza A virus (IAV) attachment. By direct stochastic optical reconstruction microscopy, confocal microscopy, and flow cytometry, we have shown that interferon-gamma reduced the size of α-2,3 and α-2,6-linked sialic acid clusters, without changing the sialic acid or epidermal growth factor receptor expression levels, or the sialic acid density within cluster on the cell surface of A549 cells. Reversing the effect of interferon-gamma on sialic acid clustering by jasplakinolide reverted the cluster size, improved IAV attachment and replication. Our findings showed the importance of sialic acid clustering in IAV attachment and infection. We also demonstrated the interference of sialic acid clustering as an anti-IAV mechanism of IFN-gamma for IAV infection.

## Highlights

• IFN-γ inhibits IAV replication
• IFN-γ reduces IAV attachment and infection by reducing sialic acid cluster size
• Reduction of sialic acid cluster size partially depends on F-actin depolymerization
• Higher sialic acid expression level does not correlate with increase IAV attachment

## Graphical abstract

**Figure:**
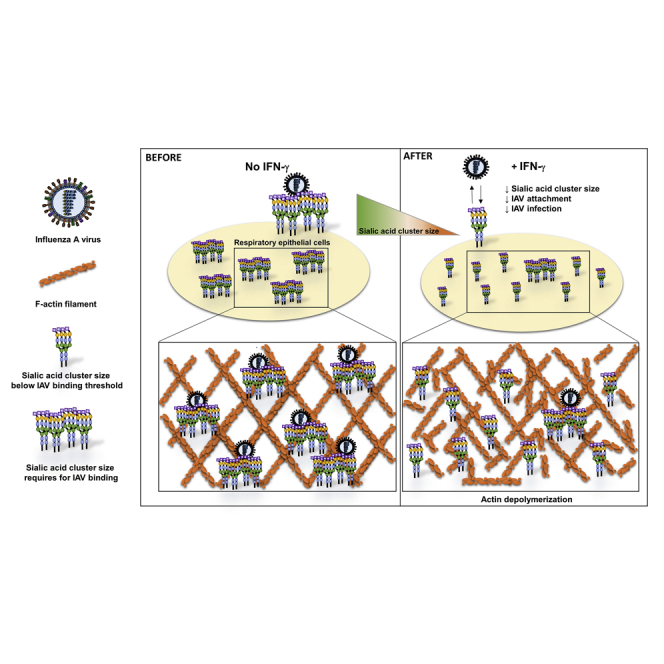

## Introduction

Influenza virus causes seasonal epidemics and pandemics which are associated with significant morbidity and mortality (Cheng et al., 2012; To et al., 2013). Pneumonia is the major complication of influenza virus infection, although extra-pulmonary complications can also occur (To et al., 2019). Both innate and adaptive immunity are crucial in limiting disease severity (Doherty et al., 2006). Genome-wide association study showed that several host genetic polymorphisms are associated with severe influenza (To et al., 2014, 2015).

The respiratory epithelium serves as the initial site of influenza A virus (IAV) replication, thereby its defense mechanisms mediated by the innate immune system provide the first line of host defense (Sanders et al., 2011). Once IAV enters into the respiratory epithelial cells, the viral RNA is recognized as foreign by pathogen recognition receptors, initiating a cascade of inflammatory immune responses (Iwasaki and Pillai, 2014; Iwasaki et al., 2017). Type I and III interferons (IFNs) are the key antiviral cytokines that are produced immediately after viral infection (Chen et al., 2018; Busnadiego et al., 2020; Vanderheiden et al., 2020). Types I/III IFNs signal through their host receptors, leading to the activation of janus kinase 1 and tyrosine protein kinase 2. The phosphorylation of signal transducer and activator of transcription 1 (STAT1) and STAT2, and their binding to interferon regulatory factor, form the interferon-stimulated gene factor 3 (ISGF3). ISGF3 then bind to interferon-stimulated response elements, leading to the activation of IFN-stimulated genes (ISGs) that limit viral replication through diverse mechanisms and create an antiviral state (Schoggins, 2019).

Unlike type I and III IFNs, IFN-γ is not secreted by respiratory epithelial cells but from immune cells, such as natural killer cells (Ge et al., 2012), natural killer T cells (Ishikawa et al., 2010), gamma-delta (γδ) T cells (Dong et al., 2018), innate lymphoid cells 1 (ILC1) (Weizman et al., 2017), and CD8^+^ T cells (Koutsakos et al., 2019). IFN-γ activates a different signaling pathway that involves a STAT1 homodimer as the downstream transcription regulator, which drives transcription of ISGs with a gamma-activated site promoter (Fensterl et al., 2015). IFN-γ can induce the ISG IFN-induced transmembrane protein 3 (IFITM3) to inhibit IAV entry by hindering the fusion of the viral envelope with the endosomal membrane, and subsequently inhibit the release of viral ribonucleoproteins (vRNP) from endosome (Brass et al., 2009; Desai et al., 2014; Feeley et al., 2011; Li et al., 2013).

Compared to the well-recognized protective roles of type I and III IFN during IAV infection, much less is known about IFN-γ and IAV infection. In patients, IFN-γ can be detected in the blood during the first 24 h of IAV infection (Bian et al., 2014; Pommerenke et al., 2012; Weizman et al., 2017). In mouse models, IFN-γ can be detected in the lung, especially from the lung resident ILC1 at 24 h.p.i (hour post infection), and ILC1 was shown to confer host protection at the initial sites of viral infection (Weizman et al., 2017; Pommerenke et al., 2012). Several groups have demonstrated that IFN-γ can protect mice during influenza virus infection (Berri et al., 2014; Weiss et al., 2010; Bot et al., 1998). Collectively, these studies have shed light on a host protective role of IFN-γ in the lung during the early stage of influenza virus infection.

In our previous study, we have shown that IFN-γ induces human tryptophanyl-tRNA synthetase to facilitate the entry of enterovirus A71 (EV-A71) (Yeung et al., 2018). Conversely, IFN-γ has also been shown to reduce human immunodeficiency virus (HIV) type 1 and hepatitis C virus infection by down-regulating the viral receptors CD4 and claudin-1 expression (Dhawan et al., 1995; Wei et al., 2009). However, the role of IFN-γ in receptor binding of IAV is not known. In current study, we investigated the role of IFN-γ during the entry phase of IAV infection in respiratory epithelial cells. We showed IFN-γ inhibited influenza A virus infection by decreasing α-2,3, α-2,6-linked sialic acid cluster size without changing the sialic acid expression levels, reduced virus attachment and replication. Further studies showed IFN-γ altered the morphology of the F-actin cytoskeleton. Inducing actin polymerization by jasplakinolide reverted the reduced α-2,6-linked cluster size, improved viral binding and replication. Our finding demonstrated the importance of sialic acid cluster size during IAV infection in lived cells, and revealed an anti-IAV mechanism of IFN-γ during the viral binding step on respiratory epithelium.

## Results

### IFN-γ inhibits influenza virus replication in respiratory epithelial cells

First, we evaluated the cellular toxicity of IFN-γ. We did not observe any toxicity for IFN-γ in A549 cells (up to 4,000 IU/mL) and Calu3 (up to 1,000 IU/mL) (Figure S1).

**Figure 1:**
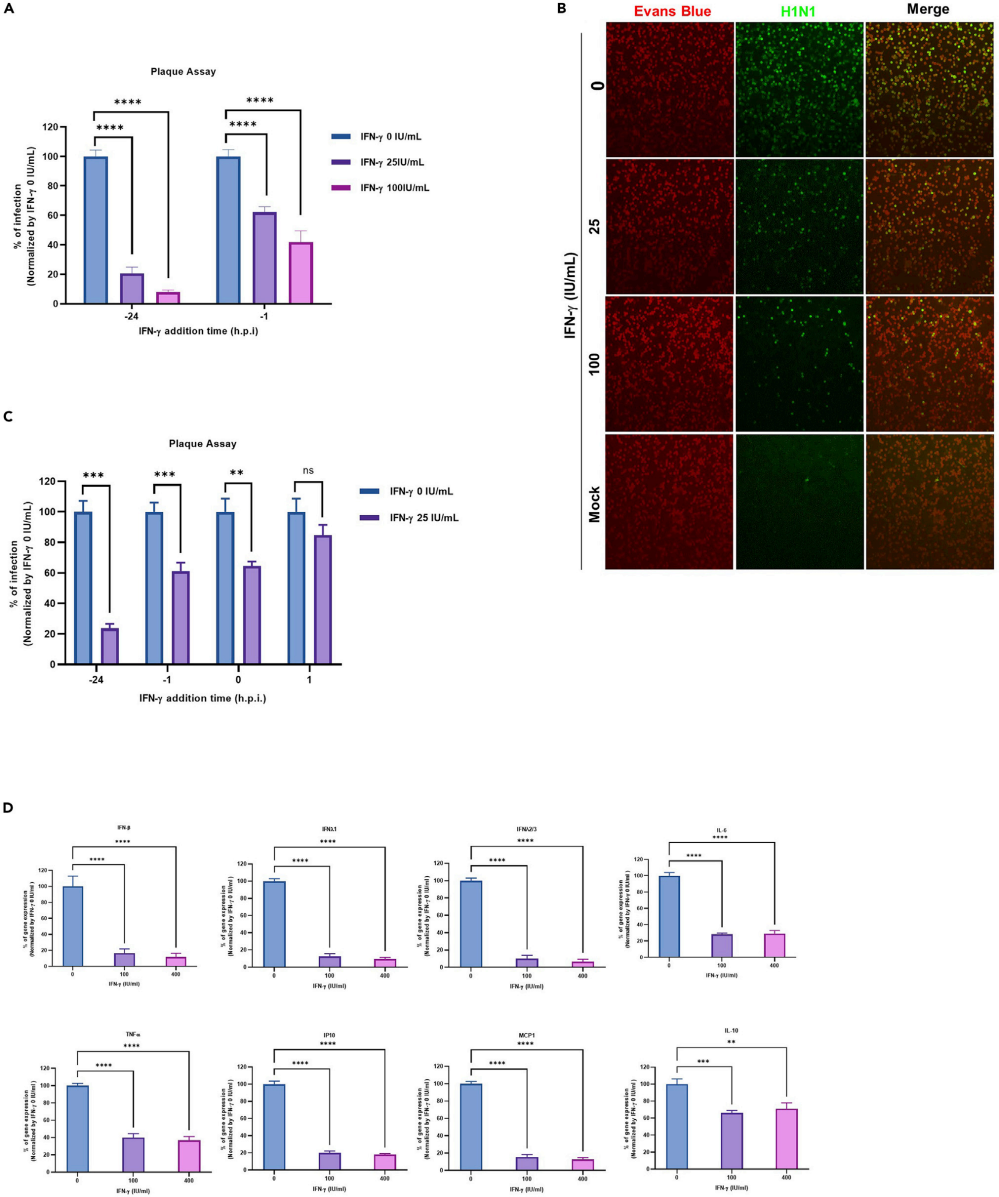
IFN-γ inhibits influenza virus replication in respiratory epithelial cells (A) A549 with or without IFN-γ pre-treatment at 1 h or 24 h were infected with A/Hong Kong/415742/2009(H1N1) virus at an MOI of 0.01. Culture supernatant was collected 24 h post infection for plaque assay with MDCK cells. The average of three independent experiments performed with three biological replicates is shown. Data are represented as mean +/− standard error of mean (SEM). Multiple t test was used to test statistical significance. ∗∗∗∗p ≤ 0.0001. Error bar indicates SEM. (B) A549 with or without 24 h IFN-γ pre-treatment wereinfected with A/Hong Kong/415742/2009(H1N1) at an MOI of 1. Infected cells were stained using the D3 Ultra 8 DFA respiratory virus screening and identification kit and viewed under epifluorescent illumination of Eurostar III plus fluorescence microscope. Magnification ×100. Representative images are from three independent experiments with three biological replicates of each condition. (C) A549 cells were pre-treated with 0 or 25 IU/mL IFN-γ at 1 h or 24 h before infection, or cells were added together with the virus at 0 h, or 1 h post viral inoculation with A/Hong Kong/415742/2009(H1N1) at an MOI of 0.01. Culture supernatant was collected 24 h post infection and tested with plaque assay in MDCK cells. The average of three independent experiments performed with three biological replicates is shown. Data are represented as mean ± SEM. Multiple t test was used to test statistical significance. ns – not significant, ∗∗p ≤ 0.01, ∗∗∗p ≤ 0.001. Error bar indicates SEM. (D) A549 cells with or without 24 h IFN-γ pre-treatment were inoculated with A/Hong Kong/415742/2009(H1N1) at an MOI of 0.1. Cell lysates were collected at 24 h.p.i for RT-qPCR to measure the expression levels of cytokines (*IFNB1*, *IFNL1*, *IFNL2/3*, *IL-6*, *TNF*, and *IL-10*) and chemokines (*CXCL10*, *CCL2*). The average of three independent experiments was performed, with two biological replicates from one experiment and three biological replicates from two experiments is shown. Data are represented as mean ± SEM Multiple t test was used to test statistical significance. ∗∗p ≤ 0.01, ∗∗∗p ≤ 0.001, ∗∗∗∗p ≤ 0.0001. Error bar indicates SEM.

Next, we evaluated the effect of IFN-γ on IAV infection in the respiratory epithelial cell line, A549. When A549 cells were pre-treated with IFN-γ for 1 h or 24 h before infection, a significant reduction of IAV replication was observed as shown in Figure 1A. To confirm this finding, we performed an immunofluorescense assay for viral protein expression (To et al., 2016). In line with the multicycle growth assay, a dose-dependent inhibition of influenza nucleoprotein expression was observed from IFN-γ pre-treated cells when compared with the un-treated cells (Figure 1B), confirming the anti-IAV effect of IFN-γ in A549 cells.

Next, we performed a time-of-addition experiment to identify the stage of viral replication that is inhibited by IFN-γ. When IFN-γ was added to the cells at 24 h and 1 h before IAV infection, IAV replication was reduced by approximately 80% and 40% (Figure 1C). The addition of IFN-γ and IAV to the cells at the same time could also reduce viral replication by 40%. However, IFN-γ did not significantly inhibit IAV replication if added 1 h.p.i. In addition to being the primary target of IAV, respiratory epithelial cells are also involved in initiating the first wave of cytokine and chemokine production to amplify the inflammatory cascades for viral clearance (Vareille et al., 2011; Denney and Ho, 2018). We thus measured the expression levels of various cytokines and chemokines by RT-qPCR. In line with the lower IAV replication, the mRNA expression levels of IAV mediated cytokines and chemokines; interferon-beta (*IFNB1*), interferon lambda 1 (*IFNL1*), interferon lambda 2/3 (*IFNL2/3*), interleukin-6 (*IL-6*), tumor necrosis factor-alpha (*TNF*), C-X-C motif chemokine ligand 10 (*CXCL10*), C-C motif chemokine ligand 2 (*CCL2*), and interlekin-10 (*IL-10*) expression were also lower in IFN-γ pre-treated cells than those of un-treated cells (Figure 1D). Collectively, these results suggest the antiviral effect was mediated before the virus enters into A549 cells.

### Cells pre-exposed to IFN-γ are protected from infectious progeny

**Figure 2:**
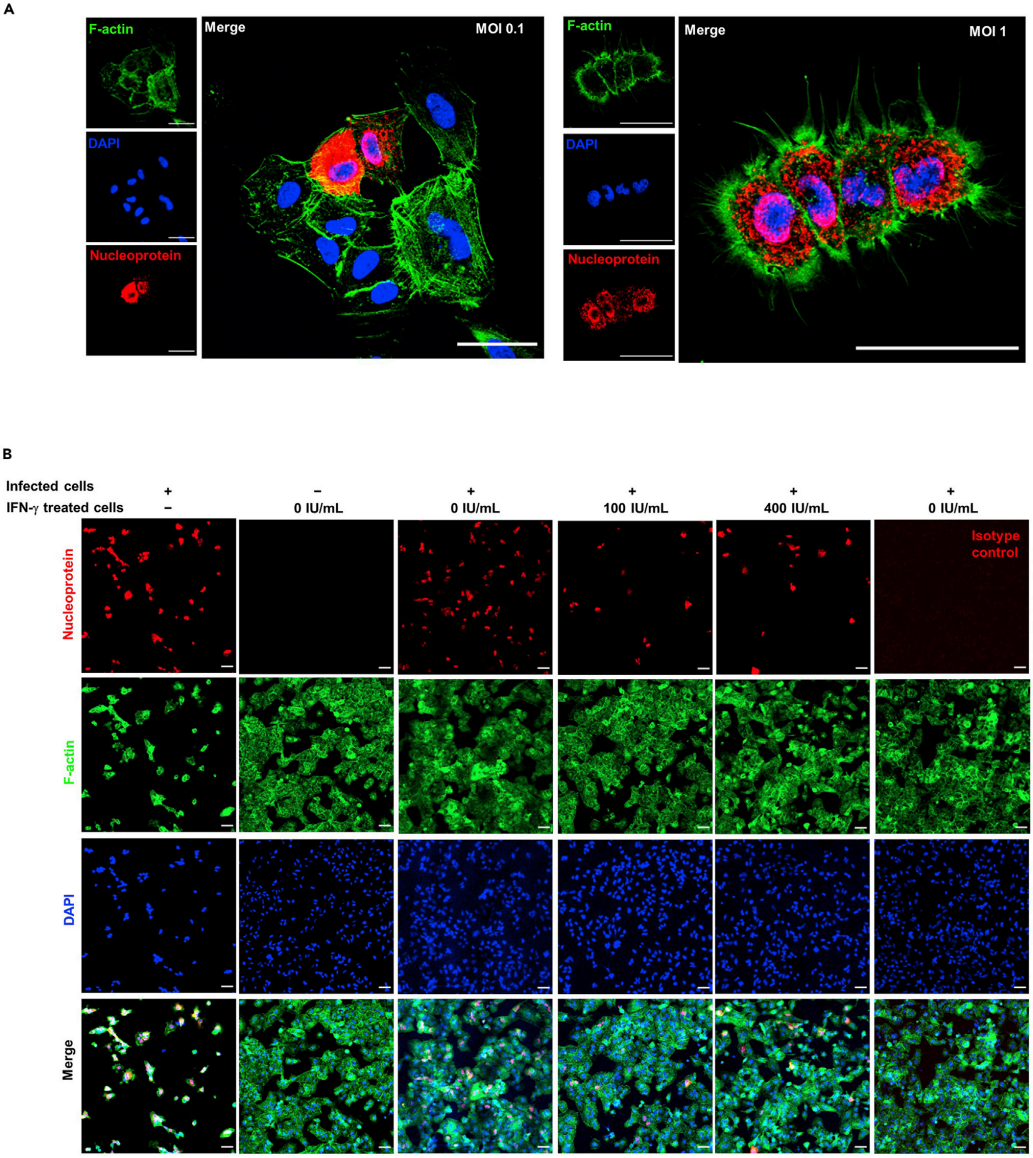
Cells pre-exposed to IFN-γ are protected from infectious progeny (A) 1 × 10^4^ A549 cells were inoculated with A/Hong Kong/415742/2009(H1N1) at an MOI of 0.1 or 1. F-actin was stained with Alexa Fluor™ 488 phalloidin (green) and IAV was stained with mouse anti-nucleoprotein IgG2a, or mouse IgG2a isotype control, and goat anti-mouse IgG AF633 (Red). All samples were mounted with Prolong™ Diamond Antifade with DAPI (Blue). Confocal images were taken with Carl Zeiss LSM880 and analyzed with software Zen 2.3 Blue Edition. Scale bar 50 μm. (B) 1 × 10^4^ A549 cells were infected with A/Hong Kong/415742/2009(H1N1) at an MOI of 1. Infected cells were co-cultured with 5 × 10^4^ of 24 h IFN-γ pre-treated, un-infected A549 cells. A total of 3 indepedent experiments were performed with image of n = 6 from each experiment.Representative confocal images are shown. F-actin was stained with Alexa Fluor™ 488 phalloidin (green) and IAV was stained with mouse anti-nucleoprotein IgG2a, or mouse IgG2a isotype control, and goat anti-mouse IgG AF633 (Red). All samples were mounted with Prolong™ Diamond Antifade with DAPI (Blue). Confocal images were taken with Carl Zeiss LSM880 and analyzed with software Zen 2.3 Blue Edition. Scale bar 50 μm.

In order to mimic the IAV spreading *in vivo*, we set up a co-culture system by mixing IAV infected and un-infected A549 cells at a ratio of 1:5. During incubation, the virus progeny from infected A549 cells will spread to non-infected A549 cells. To ensure there are enough infectious progeny secreted from the infected cells, we first assessed the virus titer required to infect over 90% of cells after 24 h in our system. At 1 multiplicity of infection (MOI), >90% of the cell population was infected (Figure 2A). Therefore, 1 MOI was selected for the viral transmission co-culture experiment.

For the co-culture experiment, non-infected IFN-treated A549 cells were much less susceptible to IAV infection when compared with mock-treated A549 cells (Figure 2B). This result suggests that pre-exposure of IFN-γ can protect cells from IAV infection.

### IFN-γ reduces IAV attachment on the cell surface

**Figure 3:**
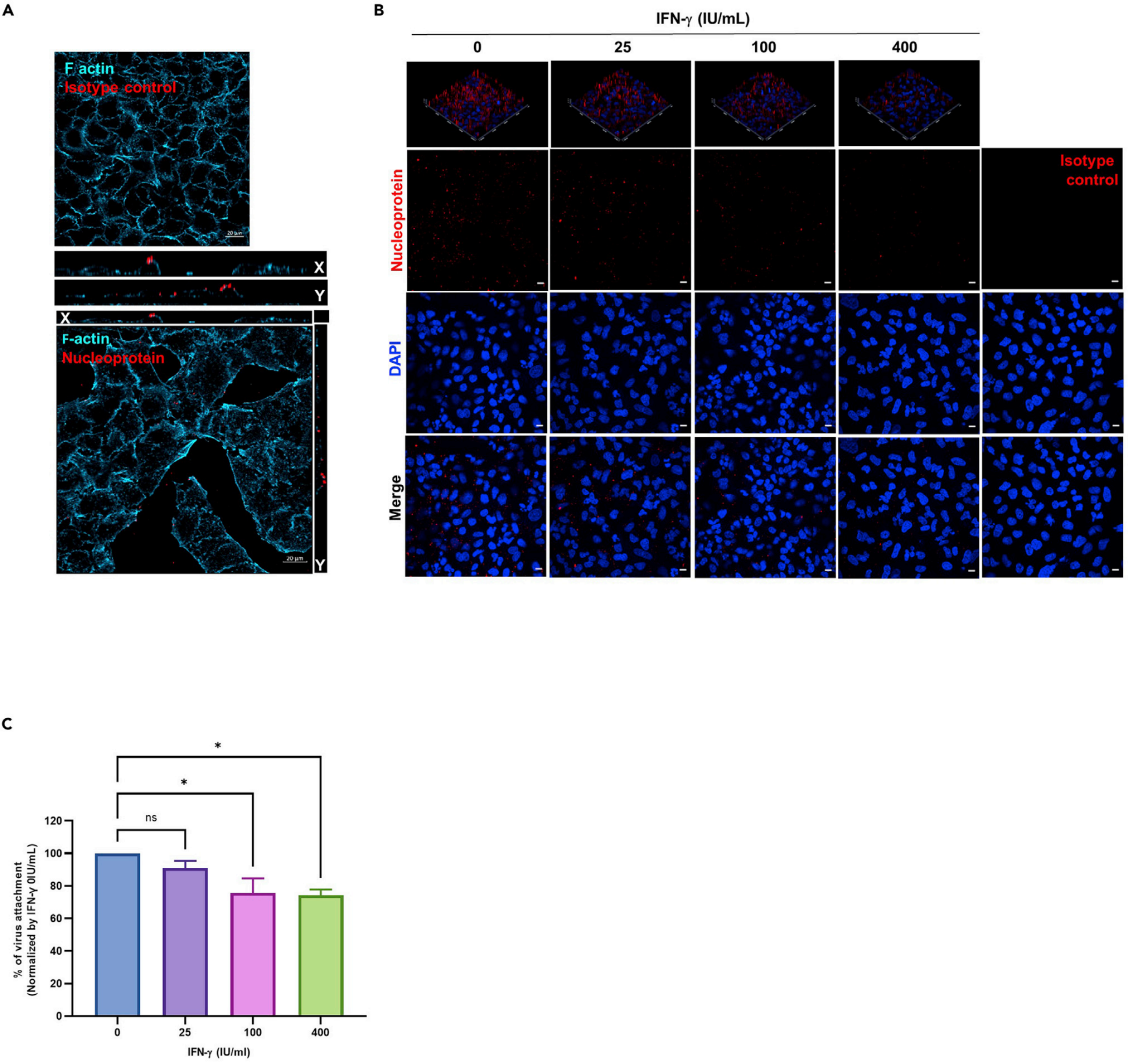
IFN-γ reduces IAV attachment on the cell surface (A and B) A549 cells were inoculated with A/Hong Kong/415742/2009(H1N1) at an MOI of 50 on ice. IAV was stained with mouse anti-nucleoprotein IgG2a or mouse IgG2a isotype control, and goat anti-mouse IgG AF633 (Red). F-actin was stained with Alexa Fluor™ 488 Phalloidin (Cyan) as indicated. All samples were mounted with Prolong™ Diamond Antifade with DAPI (Blue). Confocal images were taken with Carl Zeiss LSM710 and analyzed with software Zen 2.3 Blue Edition. (A) X-Y single-axial sections are shown to locate where the virus locates in the cell; F-actins (Cyan), nucleoprotein (Red). Scale bar 20 μm. (B) Virus attachment is shown by nucleoprotein (Red) and DAPI (Blue). A representative of confocal images is shown from 2 independent experiments of image (n = 6). Scale bar 10 μm. (C) A549 cells with or without 24 h IFN-γ pre-treatment were inoculated with A/Hong Kong/415742/2009(H1N1) at an MOI of 5 on ice. IAV was stained with rabbit anti-hemagglutinin IgG, or rabbit IgG isotype control, and donkey anti-rabbit IgG PE. Dead cells were excluded with the staining of zombie violet dye. The average of three independent experiments performed with two biological replicates is shown. Data are represented as mean ± SEM. Multiple t test was used to test statistical significance. ns - not significant, ∗p ≤ 0.05. Error bar indicates SEM.

Next, we investigated if the antiviral effect of IFN-γ is related to virus attachment. We first evaluated our attachment assay system by staining the virus and F-actin cytoskeleton on A549 cells. Confocal imaging analysis showed the X-Y single-axial section through the middle of cells, with the viruses remained on the cell surface without entering the cells (Figure 3A). By using the attachment assay system, we studied the effect of IFN-γ on IAV attachment by confocal imaging. We found a reduction of virus attachment on A549 cells pre-treated with IFN-γ than the un-treated cells (Figure 3B). To confirm this finding, we applied flow cytometry analysis as a quantitative approach. Consistent with the confocal analysis, a lower level of virus attachment was detected on cells pre-treated with IFN-γ when compared to the un-treated cells (Figure 3C). Taken together, we demonstrated that cells pre-exposed to IFN-γ have lowered IAV attachment on the cell surface.

### IFN-γ changes the morphology of α-2, 3, α-2, 6-linked sialic acids, but not their expression levels on the cell surface

**Figure 4:**
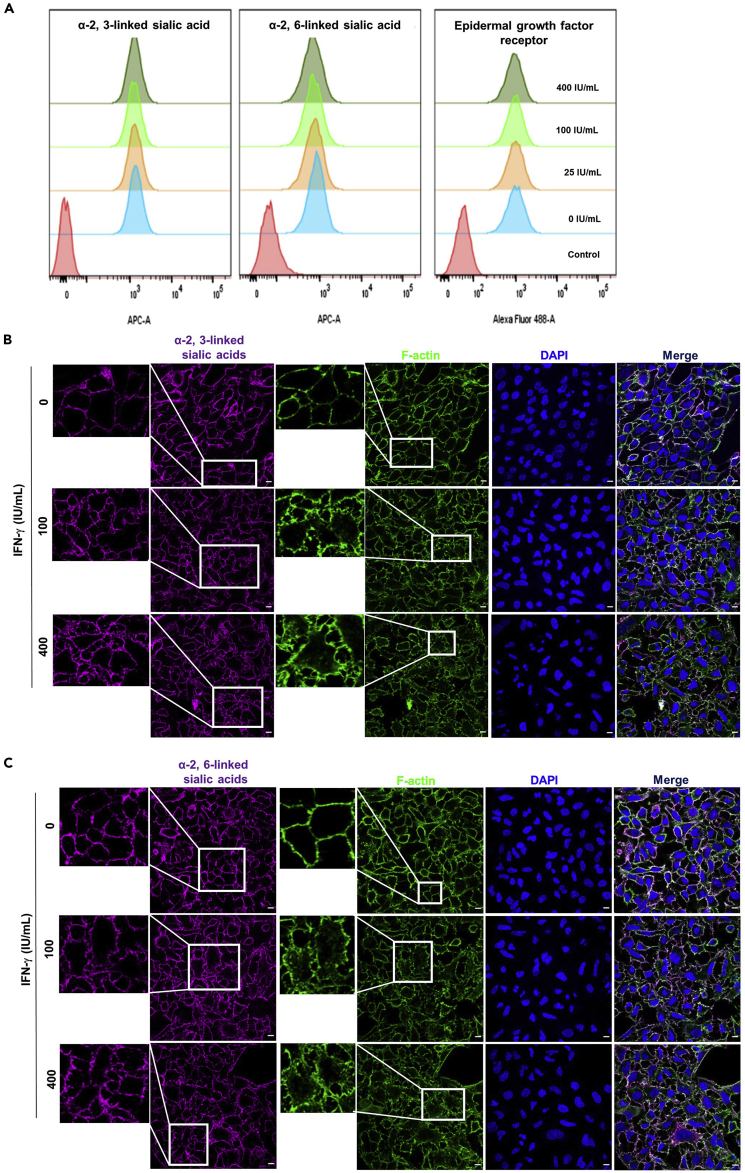
IFN-γ changes the morphology of α-2, 3-linked, α-2, 6-linked sialic acids, but not their expression levels (A) A549 cells with or without 24 h IFN-γ pre-treatment were stained with biotin-conjugated MAA for α-2, 3-linked sialic acids or biotin-conjugated SNA I for α-2,6-linked sialic acid and strep-APC or Alexa Fluor 488 mouse anti-human-EGFR IgG1. Strep-APC only and Alexa Fluor 488 anti-mouse IgG1 were used as control for sialic acids and EGFR staining. Dead cells were excluded with propidium iodide and their expression levels were analyzed with flow cytometry. Representative data are shown from three independent experiments of three biological replicates. (B and C) A549 cells with or without 24h IFN-γ pre-treatment were stained with biotin-conjugated MAA forα-2, 3-linked sialic acids or biotin-conjugated SNA I for α-2, 6-linked sialic acids together with strep-APC (Magenta). F-actin was stained with Alexa Fluor™ 488 Phalloidin (Green). All samples were mounted with Prolong™ Diamond Antifade with DAPI (Blue). Confocal images were taken with Carl Zeiss LSM 710 and analyzed with software Zen 2.3 Blue Edition. Representative images are shown from 3 independent experiments of images (n = 6). Scale bar 10 μm.

We postulated the lower IAV attachment mediated by IFN-γ is related to the expression levels of IAV attachment factor and/or receptor. Flow cytometry analysis showed IFN-γ did not affect the expression levels of α-2, 3-linked sialic acids, α-2, 6-linked sialic acids, or epidermal growth factor receptor (EGFR) (Figure 4A). We then tested the effect of IFN-γ on α-2, 6 and α-2, 3-linked sialic acid morphorolgy by confocal imaging analysis. From IFN-γ un-treated cells, α-2, 3-linked and α-2, 6-linked sialic acids distributed evenly in regular dimensions of a typical polygonal shape of epithelial-like cells. After IFN-γ pre-treatment, the distribution of α-2, 3 and α-2, 6-linked sialic acid on the cell boundary become twisted and the epithelial-like polygonal shape disappeared (Figures 4B and 4C).

One possible factor that affects the pattern of sialic acid is a change in the actin cytoskeleton. Therefore, we studied the effect of IFN-γ on F-actin filament. Similar to the sialic acid, F-actin filaments are organized in regular dimensions with defined linear filaments, creating a polygonal shape of epithelial-like cell structure. With IFN-γ pre-treatment, the linear filaments of F-actin disappeared and the linear cell boundaries became poorly defined (Figures 4B and 4C).

### IFN-γ reduces α-2,6-linked sialic acid cluster size

**Figure 5:**
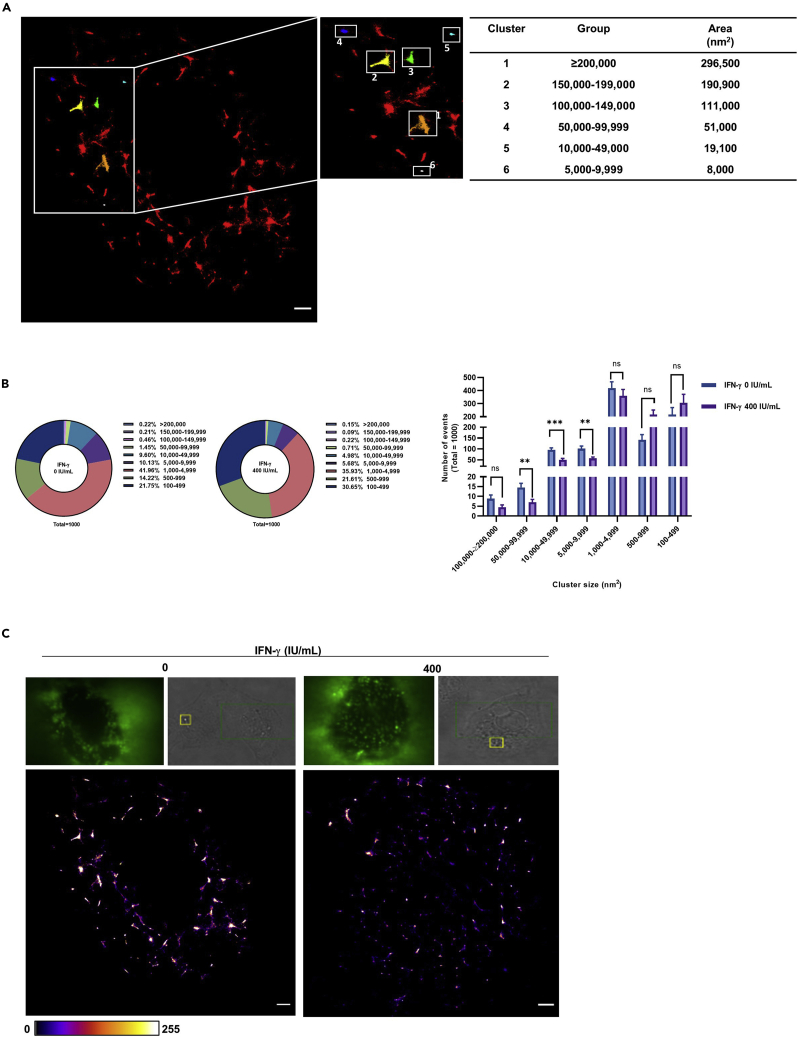
IFN-γ reduces α-2,6-linked sialic acid cluster size (A–C) A549 cells with or without 24 h IFN-γ pre-treatment were stained with biotin-conjugated SNA I, strep-AF647 and α-2,6-linked sialic acid cluster sizes were measured by dSTORM imaging. Alpha-2,6-linked sialic acid cluster with different sizes was identified by image-based analysis from the built in function of Particle Analysis in Image J, Fiji, with a fixed threshold setting of 32/255. Four independent experiments with an average of n = 6 cells were performed. (A) Identification of α-2,6-linked sialic acid cluster with different size; area (nm^2^). Scale bar 1 μm. (B) The top 1000 with the largest cluster sizes were categorized into 9 groups; 1) ≥200,000 nm^2^ 2) 150,000–199,999 nm^2^ 3) 100,000–149,999 nm^2^ 4) 50,000–99,999 nm^2^ 5) 10,000–49,999 nm^2^ 6) 5,000–9,999 nm^2^ 7) 1,000–4,999 nm^2^ 8) 500–999 nm^2^ 9) 100–499 nm^2^. The number of event in percentage is presented as pie chart and the statistical significance of 4 independent experiments (an average of n = 6 cells) is shown as bar chart. Data are represented as mean ± SEM. Multiple t test was used to test statistical significance. ns - not significant, ∗∗p ≤ 0.001, ∗∗∗p ≤ 0.001. Error bar indicates SEM. (C) Representative images of - α2,6-linked sialic acid clusters on un-treated and 24 h IFN-γ-treated A549 cells are shown. Scale bar 1 μm.

The affinity of single glycan–HA is relatively weak with dissociation constant (K_d_) in millimolar range (Sauter et al., 1989, 1992; Sieben et al., 2012). The weak binding force suggests stable binding between sialic acids and HA is avidity driven. Thus, sialic acid cluster size could be a criterion for stable IAV binding. We hypothesize that the reduced virus attachment correlates with the receptor cluster size for stable viral binding. By direct stochastic optical reconstruction microscopy (dSTORM) analysis, we found that α-2,6-linked sialic acid forms heterogenous clusters with different cluster sizes on the cell surface (Figure 5A).

Next, we assessed the cluster size of cell surface α-2,6-linked sialic acid using the particle analysis function of software Image J (Fiji) (Schindelin et al., 2012; Henriques et al., 2010). The particle analysis function is an image-based method to identify qualified clusters after fixed threshold setting. An example of identified cluster with different size is shown in Figure 5A. From the identified clusters, we selected the 1000 largest clusters for data analysis. Among the 9 cluster groups analyzed, the clusters with size between 50,000 and 99,999 nm^2^ (control: 1.45%; IFN-γ pre-treated cells: 0.71%), 10,000–49,999 nm^2^ (control 9.6%; IFN-γ pre-treated cells 4.98%), and 5,000–9,999 nm^2^ (control 10.13%; IFN-γ pre-treated cells 5.68%), were significantly reduced by IFN-γ (Figures 5B and 5C).

### The anti-influenza A virus effect of IFN-γ is partially dependent on actin depolymerization

**Figure 6:**
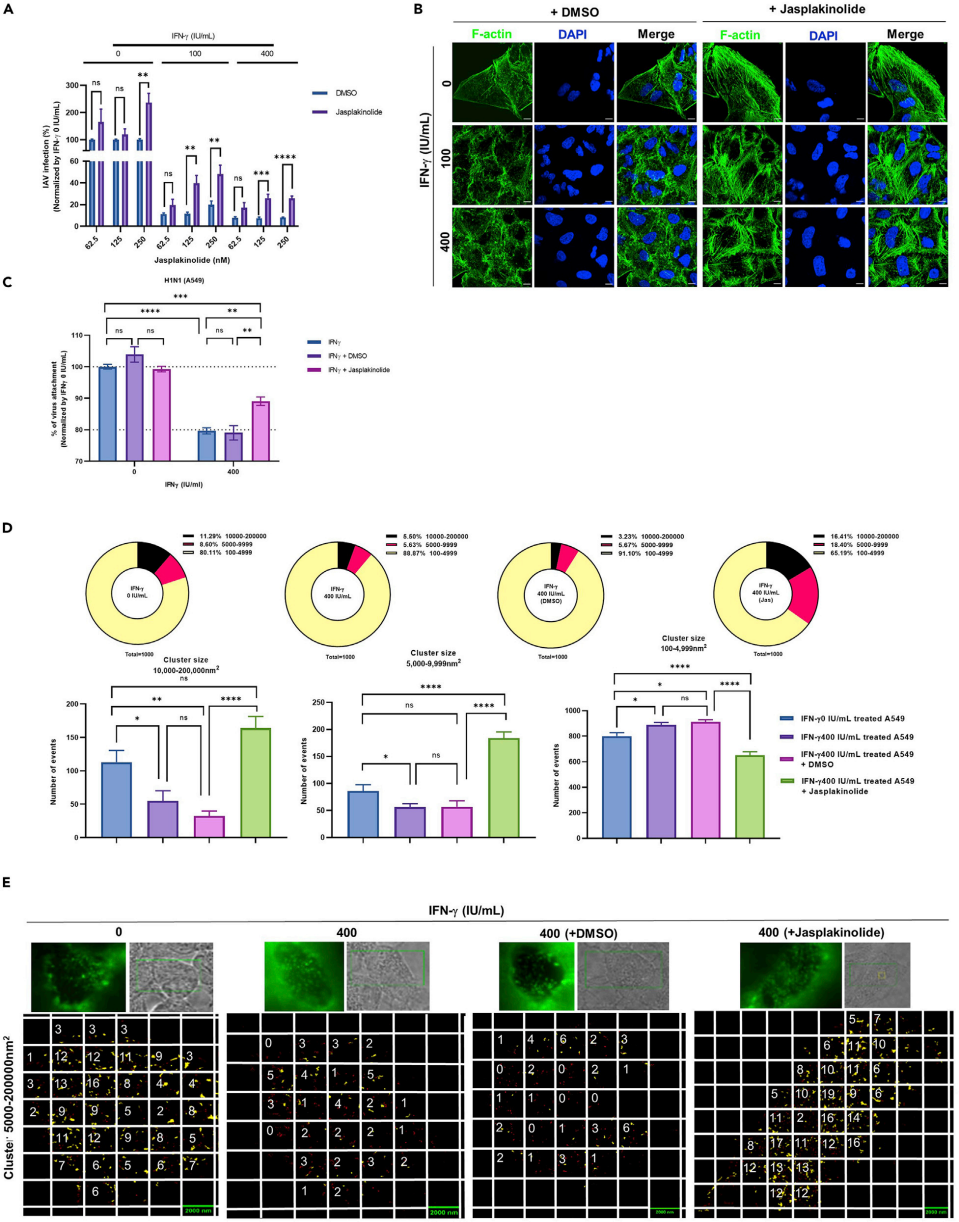
The anti-influenza A virus effect of IFN-γ is partially dependent on actin depolymerization (A)A549 cells with or without 24 h IFN-γ pre-treatment were treated with 62.5, 125, and 250 nM of jasplakinoline or the equivalent dilution of DMSO at 37°C. Cells were inoculated with A/Hong Kong/415742/2009(H1N1) at an MOI of 0.01. Cell culture supernatant was collected at 24 h post infection for virus purification; M gene was quantified by RT-qPCR. The average of two independent experiments performed with three biological replicates is shown. Data are represented as mean ± SEM. Multiple t test was used to test statistical significance. ns - not significant, ∗∗p ≤ 0.001, ∗∗∗p ≤ 0.001, ∗∗∗∗p ≤ 0.0001. Error bar indicates SEM. (B) A549 cells with or without 24 h IFN-γ pre-treatment were treated with 125 nM of jasplakinolide or the equivalent dilution of DMSO at 37°C. F-actin was stained with Alexa Fluor™ 488 Phalloidin (Green). All samples were mounted with Prolong™ diamond Antifade with DAPI (Blue). Confocal images were taken with Carl Zeiss LSM880 and analyzed with software Zen 2.3 Blue Edition. Representative images are shown from two independent experiments of images (average of n = 6). Scale bar = 10 μm. (C) A549 cells with or without 24 h IFN-γ pre-treatment were treated with 125 nM jasplakinolide or DMSO at 37°C and inoculated with A/Hong Kong/415742/2009(H1N1) at an MOI of 5 at 4°C. IAV were stained with rabbit anti-hemagglutinin IgG or rabbit IgG isotype control, and donkey anti-rabbit IgG PE. Dead cells were excluded with the staining of zombie violet dye. The average of two independent experiments performed with three biological replicates is shown. Data are represented as mean ± SEM. Multiple t test was used to test statistical significance. ns - not significant, ∗∗p ≤ 0.001, ∗∗∗p ≤ 0.001, ∗∗∗∗p ≤ 0.0001. Error bar indicates SEM. (D) A549 cells pre-treated with 24 h IFN-γ were treated with 125 nM of jasplakinolide or DMSO at 37°C. Alpha-2,6-linked sialic acid was stained with biotin-conjugated SNA I and strep-AF647 and α-2,6-linked sialic acid cluster size was analyzed by dSTORM imaging. The top 1000 clusters with the largest size were selected and divided into 3 groups; 10,000->200,000 nm^2^, 5,000–9,999 nm^2^, and 100–4,999 nm^2^. The number of event in percentage is presented as pie chart and the statistical significance of the average of two independent experiment of (n = an average of 6 cells) is shown as bar chart. Data are represented as mean ± SEM. Multiple t test was used to test statistical significance. ns - not significant, ∗p ≤ 0.05, ∗∗p ≤ 0.001, ∗∗∗∗p ≤ 0.0001. Error bar indicates SEM. (E) dSTORM analysis of clusters size of 5,000-200,000 nm^2^ are highlighted in yellow. A fixed area of 4 μm^2^ (2 × 2 μm) are shown as squares. The number of cluster within the area of 4 μm^2^ is counted. Two independent experiment of an average of 6 cells was performed and the representative images are shown. Scale bar = 2 μm.

It was reported that IFN-γ shortens actin filament by guanylate-binding protein 1 (GBP-1) induction (Ostler et al., 2014). We thus tested if jasplakinolide, an actin polymerization inducer (Bubb et al., 1994, 2000), could reverse the anti-IAV effect of IFN-γ. Jasplakinolide reversed the antiviral effect of IFN-γ at doses of 125 nM and 250 nM (Figure 6A). Furthermore, we found that 250 nM of jasplakinolide also increased IAV replication in cells without IFN-γ pre-treatment. As such, to avoid the background effect caused by jasplikinolide, we selected 125-nM jasplikinolide treatment for subsequent experiments.

Next, we checked the morphology of F-actin filament by confocal imaging. From IFN-γ pre-treated cells, we found jasplakinolide treatment restored the linear filament morphology as observed in IFN-γ un-treated cells. Furthermore, we also found a higher number of linear F-actin filaments from the jasplakinolide-treated cells (Figure 6B).

To evaluate if inducing actin polymerization can reverse the inhibitory effect of IFN-γ on IAV attachment, cells were treated with 125 nM jasplakinolide or DMSO and IAV attachment was measured by flow cytometer. FACS analysis showed neither DMSO nor jasplakinolide has any effect on IAV attachment from IFN-γ un-treated cells. Jasplakinolide reduced the inhibitory effect of IFN-γ by 50% (Figure 6C).

We next studied the impact of jasplakinolide on the effect of IFN-γ on α-2,6-linked sialic acid cluster size. IFN-γ reduced the event numbers of cluster 10,000–200,000 nm^2^ and 5,000–9,999 nm^2^ by approximately two folds (Figure 6D). Jasplakinolide treatment completely reverted the cluster size of α-2,6-linked sialic acid from IFN-γ pre-treated cells by increasing the event numbers of cluster 10,000–200,000 nm^2^ and 5,000–9,999nm^2^ by approximately three to four folds (Figure 6D). The increase of cluster number is also accompanied with higher cluster density throughout the cell membrane (Figure 6E). Taken together, we showed that jasplakinolide restored the effect of IFN-γ on F-actin filament morphology, α-2,6-linked sialic acid cluster size, and improved IAV attachment and replication.

### IFN-γ and jasplakinolide have no effect on the density of α-2,6-linked sialic acid clusters

**Figure 7:**
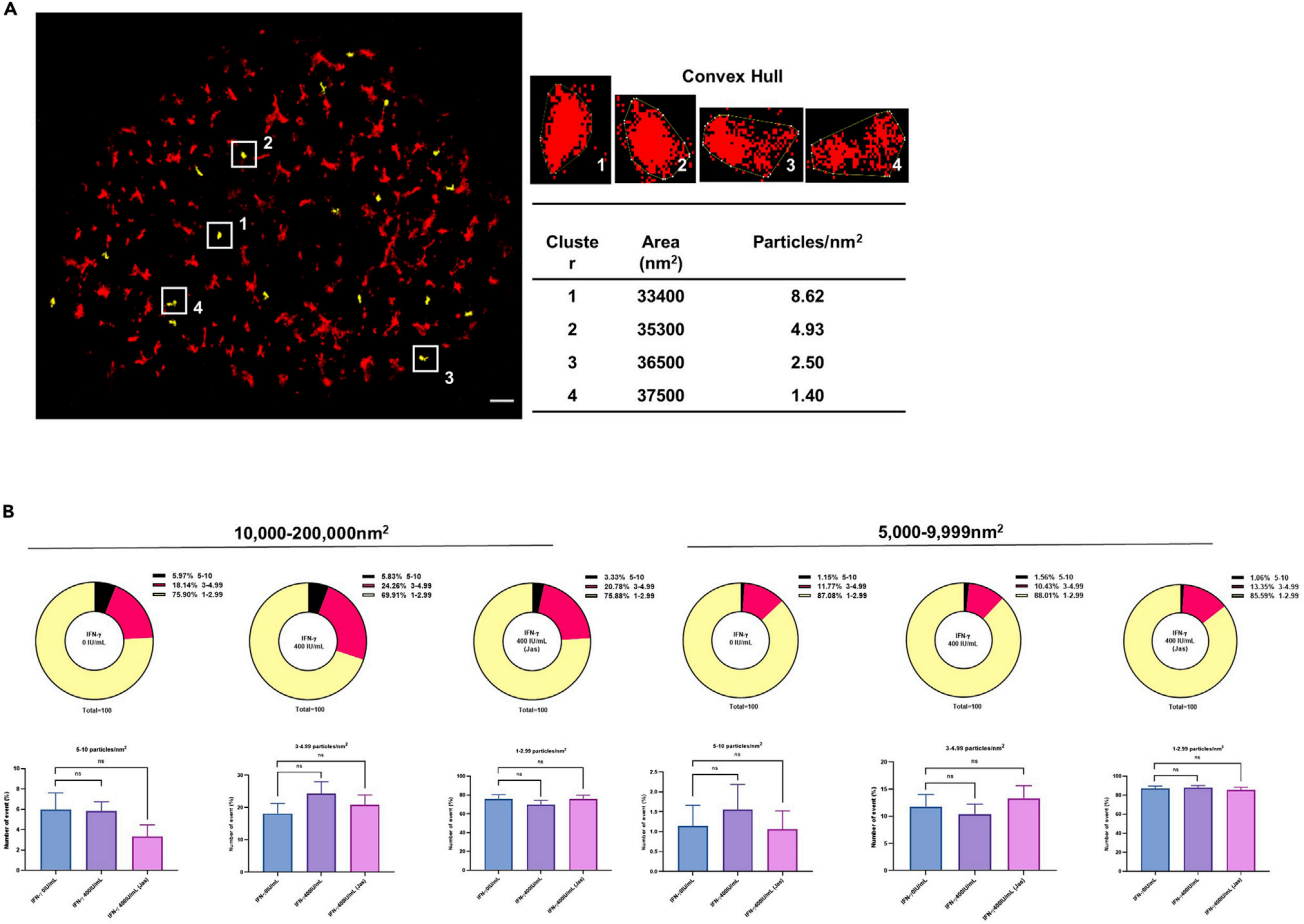
IFN-γ and jasplakinolide have no effect on the density of α-2,6-linked sialic acid clusters (A) The longest diagonal of the convex hull polygons around the clusters was identified by Image J, Fiji. Clusters with size 30,000–39,000 nm^2^ are highlighted in yellow. The α-2,6-linked sialic acid densities were calculated from the number of α-2,6-linked sialic acid particle localizations observed within an area described by convex hull of the cluster. Scale bar = 1 μm. (B) The number of α-2,6-linked sialic acid particle within 10,000–200,000 nm^2^ and 5,000–9,999 nm^2^ clusters are shown. The percentages of 1–2.99, 3–4.99, and 5–10 α-2,6-linked sialic acid particles/nm^2^ are shown in pie chart. Statistical significance of the average from two independent experiments with an average of n = 6 cells is shown as bar chart. Results were obtained from the same experiment of Figure 6D with further analysis. Data are represented as mean ± SEM. Multiple t test was used to test statistical significance. ns - not significant. Error bar indicates SEM.

We next analyzed the density of α-2,6-linked sialic acid per cluster. The density is defined as the number of sialic acid particles per cluster. Images were analyzed with Fiji with workflow as follow. Localizations from dSTORM were plotted into particle distribution histogram with QuickPALM plugin (Henriques et al., 2010). Clusters are defined when there are at least 2 localizations in a 100 nm^2^ space. Alpha-2,6-linked sialic acid densities were then calculated from number of localizations observed within an area described by convex hull of the cluster. Clusters were classified according to their area and sialic acid densities (Figure 7A). Our results show there were no significant differences in the density of α-2,6-linked sialic acid per cluster between IFN-γ un-treated cells and IFN-γ pre-treated cells with or without jasplakinolide, indicating the density of sialic acid per cluster has no impact on IAV infection (Figure 7B).

### IFN-γ reduces H3N2 and H5N1 replication, attachment, and 2,3-linked sialic acid cluster size in A549 cells

**Figure 8:**
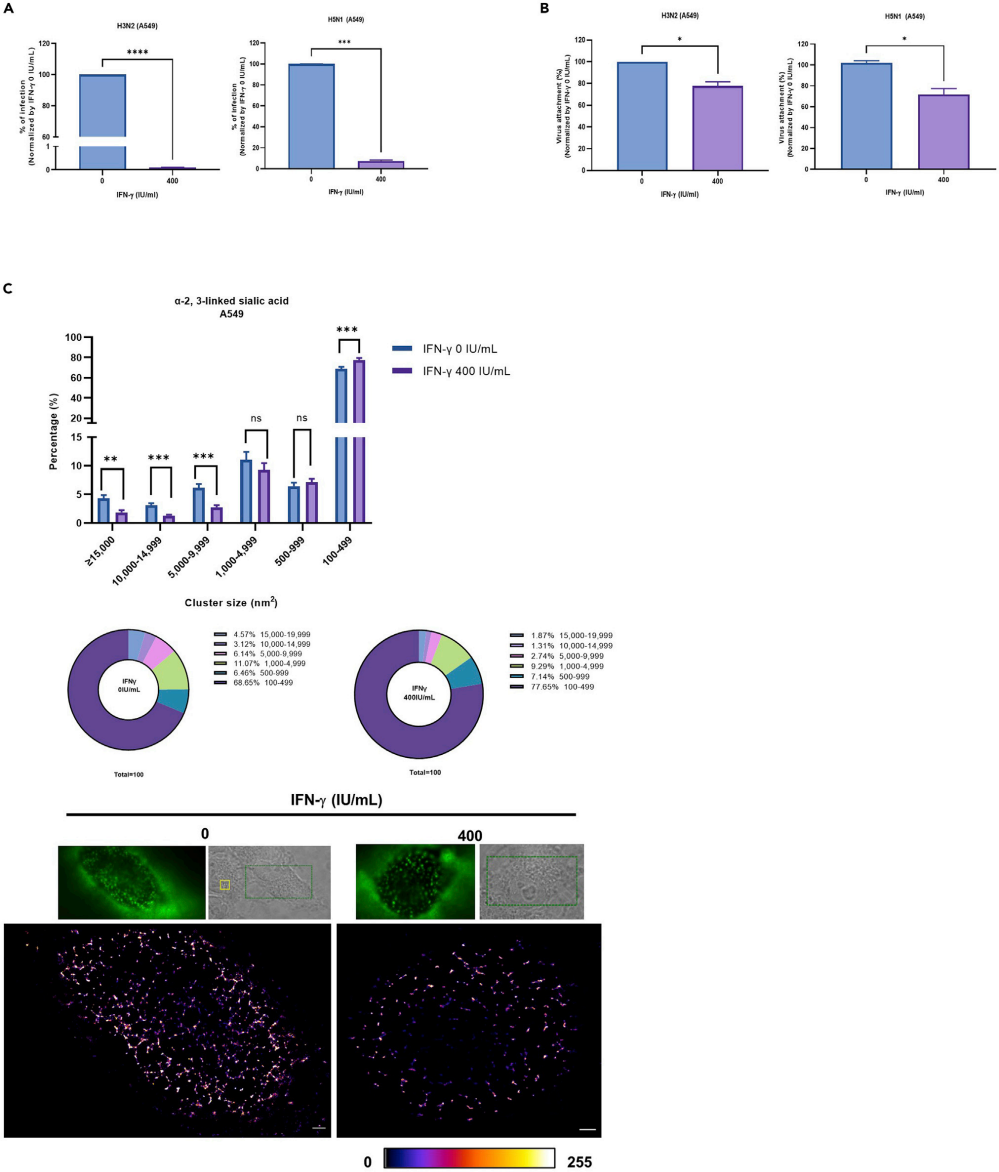
IFN-γ reduces H3N2 and H5N1 replication, attachment, and 2,3-linked sialic acid cluster size in A549 cells (A) A549 cells were pre-treated with 0 or 400 IU/mL IFN-γ for 24 h before inoculation with A/Hong Kong/417610/2018 (H3N2) or A/Vietnam/1194/2004 (H5N1) at an MOI of 0.1. Culture supernatant was collected 24 h post infection and tested with plaque assay in MDCK cells. The average of two independent experiments performed with two biological replicates is shown. Data are represented as mean ± SEM. Unpaired t test was used to test statistical significance. ∗∗∗p ≤ 0.001, ∗∗∗∗p ≤ 0.0001. Error bar indicates SEM. (B) A549 cells with or without 24 h IFN-γ pre-treatment were inoculated with A/Hong Kong/417610/2018 (H3N2) at an MOI of 5 or A/Vietnam/1194/2004 (H5N1) at an MOI of 1. For H3N2, IAV was stained with influenza A H3N2 HA antibody, rabbit monoclonal antibody or isotype control rabbit IgG, and donkey anti-rabbit IgG PE. Dead cells were excluded with the staining of zombie violet dye. For H5N1, cells were washed three times with PBS and lysed with RLT buffer. Cell lysates were collected to measure M gene by RT-qPCR. The average of two independent experiments performed with three biological replicates is shown. Data are represented as mean ± SEM. Unpaired t test was used to test statistical significance. ∗p ≤ 0.05. Error bar indicates SEM. (C) dA549 cells with or without 24 h IFN-γ pre-treatment were stained with biotin-conjugated MAA and strep-AF647. Alpha-2,3-linked sialic acid cluster sizes were measured by dSTORM imaging and analyzed by image-based analysis from the built in function of Particle Analysis in Image J, Fiji, with a fixed threshold setting of 85/255. The cluster sizes were categorized into 6 groups; 1) ≥15,000 nm^2^ 2) 10,000–14,999 nm^2^ 3) 5,000–9,999 nm^2^ 4) 1,000–4,999 nm^2^ 5) 500–999 nm^2^ 6) 100–499 nm^2^. The number of event in percentage is presented as pie and bar chart. The statistical significance of the average of 2 independent experiments with an average of n = 8 cells is shown. Data are represented as mean ± SEM. Multiple t test was used to test statistical significance. ns - not significant, ∗p ≤ 0.05, ∗∗p ≤ 0.01, ∗∗∗p ≤ 0.001, ∗∗∗∗p ≤ 0.0001. Error bar indicates SEM. Representative images of α-2,3-linked sialic acid clusters on un-treated and IFN-γ--treated A549 cells are are shown. Scale bar 1 μm.

To extend our study to other influenza strains, we repeated the experiments with H3N2 and H5N1 in A549 cells. As observed with H1N1, the viral replication (Figure 8A) and attachment (Figure 8B) were also reduced with H3N2 and H5N1 in IFN-γ pre-treated cells. Furthermore, dSTORM analysis showed IFN-γ pretreatment reduced α-2,3-linked sialic acid cluster size in A549 cells (Figure 8C). Taken together, these studies showed the antiviral effects of IFN-γ that observed with H1N1 is also applied to other IAV subtypes.

### IFN-γ reduces IAV replication, attachment, and sialic acid cluster size, but increases the sialic acid expression level in Calu3 cells

**Figure 9:**
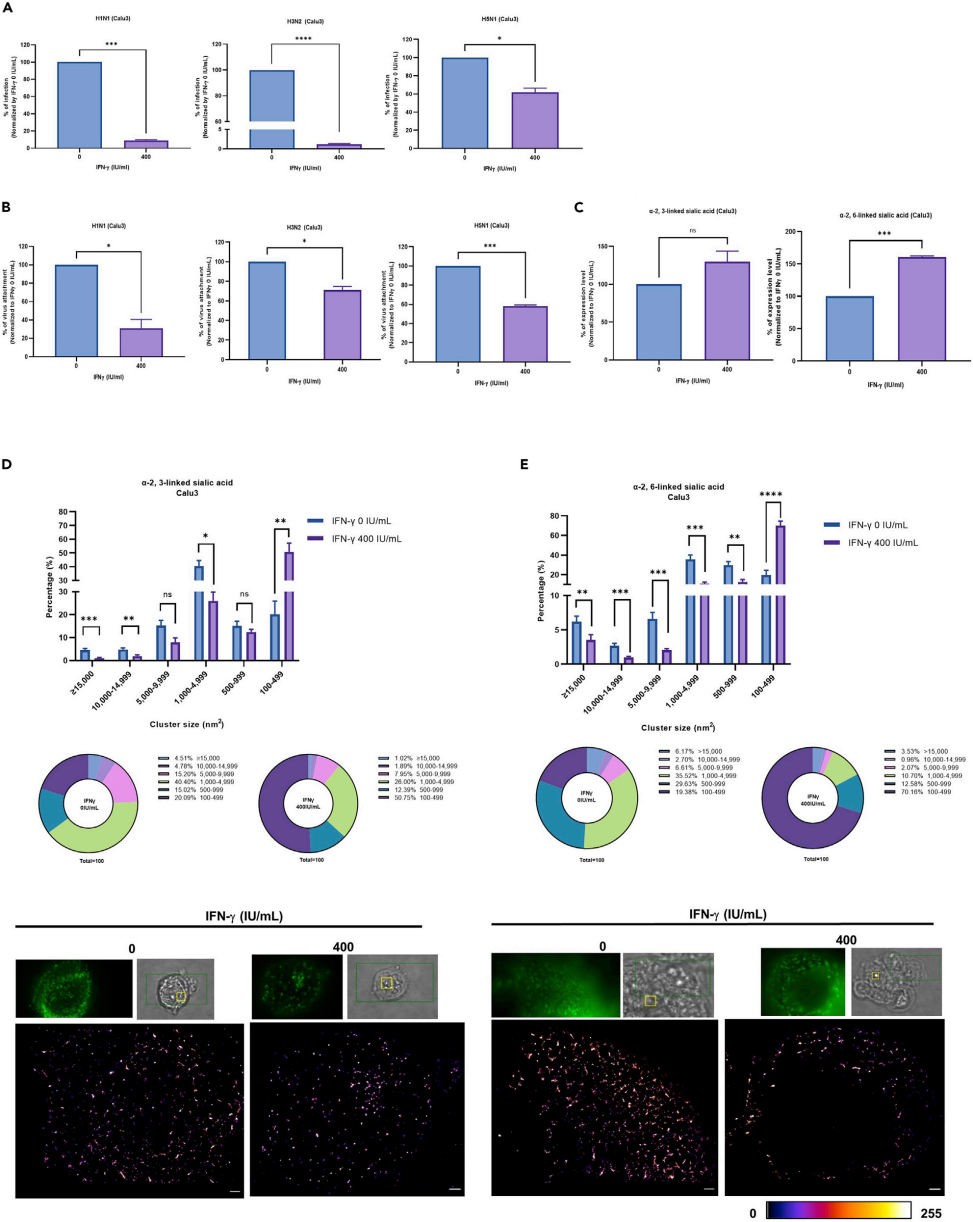
IFN-γ reduces IAV replication, attachment and sialic acid cluster size, but increases sialic acid expression level in Calu3 cells (A) Calu3 cells were pre-treated with 0 or 400 IU/mL IFN-γ for 24 h before inoculation with A/Hong Kong/415742/2009 (H1N1) or A/Hong Kong/417610/2018 (H3N2) or A/Vietnam/1194/2004 (H5N1) at an MOI of of 0.1. Culture supernatant was collected 24 h post infection and tested with plaque assay in MDCK cells. The average of two independent experiments performed with three biological replicates is shown. Data are represented as mean ± SEM. Unpaired t test was used to test statistical significance. ∗p ≤ 0.05, ∗∗∗p ≤ 0.001, ∗∗∗∗p ≤ 0.0001. Error bar indicates SEM. (B) Calu3 cells with or without 24 h IFN-γ pre-treatment were inoculated with A/Hong Kong/415742/2009 (H1N1), or A/Hong Kong/417610/2018 (H3N2) at an MOI of 5 or A/Vietnam/1194/2004 (H5N1) at an MOI of 1 at 4°C. For H1N1 and H3N2, IAV was stained with anti-hemagglutinin rabbit monoclonal antibody or isotype control rabbit IgG and donkey anti-rabbit IgG PE. Dead cells were excluded with the staining of zombie violet dye. For H5N1, cells were washed three times with PBS and lysed with RLT buffer. Cell lysates were collected to measure M gene by RT-qPCR. The average of two independent experiments performed with three biological replicates is shown. Data are represented as mean ± SEM. Unpaired t test was used to test statistical significance. ∗p ≤ 0.05, ∗∗∗p ≤ 0.001. Error bar indicates SEM. (C) Calu3 cells with or without 24 h IFN-γ pre-treatmentwere stained with biotin-conjugated MAA for α-2, 3-linked sialic acids or biotin-conjugated SNA I for α-2,6-linked sialic acid together with strep-APC. Dead cells were excluded with zombie violet dye and their expression levels were analyzed with flow cytometry. The average of two independent experiments with three biological replicates is shown. Data are represented as mean ± SEM. Unpaired t test was used to test statistical significance. ns - not significant, ∗∗∗p ≤ 0.001. Error bar indicates SEM. (D and E) Calu3 cells with or without 24 h IFN-γ pre-treatment were stained with biotin-conjugated MAA or biotin-conjugated SNA I and strep-AF647. Alpha-2,3- and α-2,6-linked sialic acid cluster sizes were measured by dSTORM imaging and analyzed by image based analysis from the built in function of Particle Analysis in Image J, Fiji, with a fixed threshold setting of 85/255. The cluster sizes were categorized into 6 groups; 1) ≥15,000 nm^2^ 2) 10,000–14,999 nm^2^ 3) 5,000–9,999 nm^2^ 4) 1,000–4,999 nm^2^ 5) 500–999 nm^2^ 6) 100–499 nm^2^. The average of two independent experiments with an average of n = 8 cells is shown as bar and pie charts. Multiple t test was used to test statistical significance. ns - not significant, ∗p ≤ 0.05, ∗∗p ≤ 0.001, ∗∗∗p ≤ 0.001, ∗∗∗∗p ≤ 0.0001. Error bar indicates SEM. Representative images of α-2,3- and α-2,6-linked sialic acid clusters on un-treated and IFN-γ-treated Calu3 cells are shown. Scale bar 1 μm.

We next extend our studies with another respiratory epithelial cell, Calu3. Consistent with A549 cells, H1N1, H3N2, and H5N1 replication (Figure 9A), attachment (Figure 9B), together with α-2,6 and α-2,3-linked sialic acid cluster size (Figures 9D and 9E) were all reduced in IFN-γ pre-treated Calu3 when compared to the untreated cells. Interestingly, we found the α-2,6-linked sialic acid expression level is higher in Calu3 cells with IFN-γ pretreatment when compared to the control cells (Figure 9C). This is in line with A549, in which we found no difference in the sialic acid expression level between A549 cells with or without IFN-γ pretreatment (Figure 4C), but the attachment of H1N1, H3N2, and H5N1 was reduced (Figures 3B, 3C, and 8B). Taken together, these data suggested that higher sialic acid expression level is not sufficient to influence IAV binding.

## Discussion

IFNs are well recognized for their anti-IAV activities. Much effort has been focused on type I and III IFNs due to their rapid anti-IAV protective effects on respiratory epithelial cells, while the implication of type II IFN, IFN-γ, remain poorly understood. Previous studies showed that IFN-γ can mediate cell-directed antiviral effects by regulating the expression levels of cell surface receptors or inducing IFITM3 to inhibit viral and endosomal membrane fusion (Dhawan et al., 1995; Wei et al., 2009; Brass et al., 2009). In the current study, we demonstrated that instead of affecting the expression of IAV receptors, IFN-γ reduced the cluster size of sialic acid on the host cells, which impairs IAV binding onto host cells and infection. Mechanistically, we showed the inhibitory effect of IFN-γ on sialic acid clustering depends on actin depolymerization. Together, we have discovered an anti-IAV mechanism of IFN-γ by reducing IAV binding onto respiratory epithelial cells through interfering sialic acid clustering.

Viral binding has a crucial impact for viral entry and replication. Several receptors/attachment/entry factors have been reported, such as sialyated glycoprotein (Nicholls et al., 2007), nucleolin (Chan et al., 2016), EGFR (Eierhoff et al., 2010), dendritic cell-specific intercellular adhesion molecule-3-grabbing nonintegrin (Gillespie et al., 2016), and macrophage mannose receptor (Reading et al., 2000). The sialyated glycoprotein is the most important receptor for IAV. Different sialic acid and particular structural sialic acid conformation have been reported as the determinants for IAV binding and infection (Chandrasekaran et al., 2008; Suzuki et al., 2000). In the current study, we have shown that cluster size is an important determinant for IAV binding. dSTORM and FACS analysis have shown that the antiviral effect of IFN-γ reduces viral attachment and sialic acid cluster size, whereas the sialic acid density within cluster or sialic acid expression level has no implication. Our result is in line with the idea on multivalent bindings proposed (Sauter et al., 1989, 1992). Further evidence was obtained from a virus-binding simulation study, showing a positive correlation between sialic acid clustering and IAV mobility (Sieben et al., 2020). Our current study advances previous understanding on virus-glycan interaction by demonstrating the importance of sialic acid cluster size, but not sialic acid density within cluster or sialic acid expression level could influence IAV binding on respiratory epithelial cells. Therefore, the determining factor for influenza virus-receptor binding depends on the sialic acid species, structural conformation, and sialic acid cluster size.

We have shown IFN-γ pre-treatment could change the F-actin cytoskeleton (Figure 6B), which concurs with the findings of other studies (Paul et al., 2012). It has been shown that IFN-γ can shorten actin filament by GTPase guanylate-binding protein 1 (GBP-1) induction (Ostler et al., 2014). Inducing actin polymerization by jasplakinolide not only restores the morphology of linear F-actin filament but also the sialic acid cluster size (Figures 6B and 6D). Therefore, our results suggest that the inhibitory effect of IFN-γ on sialic acid clustering is mediated by actin depolymerization.

Although jasplakinolide treatment completely reverted the inhibitory effect of IFN-γ on α-2,6-linked siliac acid cluster size, IAV attachment and replication were not completely recovered (Figures 6C and 6A). There are several possibilities. First, IAV infection involves sialic acid for binding and EGFR activation for entry (Eierhoff et al., 2010). It has been shown that IFN-γ inhibited EGFR tyrosine phosphorylation (Paul et al., 2012), which could inhibit IAV entry and infection (Eierhoff et al., 2010). Second, different types of actin filament assembly can affect the cytoskeleton structure (Suarez and Kovar, 2016). For example, human actin-related protein 2/3 (Arp2/3) protein complex induces the branching of F-actin network. This protein complex is composed of seven subunits, ARP2, ARP3, ARPC1, ARPC2, ARPC3, ARPC4, and ARPC5 (Goley and Welch, 2006). It has been shown that IFN-γ pre-treated A549 cells have lower ARPC2 and ARPC1A mRNA expression levels when compared to the untreated cells (Sanda et al., 2006). Our confocal microscopy analysis suggests that although jasplakinolide restores the linear filaments of actin, the morphology of the actin cytoskeleton was still different from the IFN-γ untreated cells (Figure 6B). Therefore, inducing actin polymerization by jasplakinoline may not be sufficient to reconstruct the complex organization of actin cytoskeleton back to its native form and affect IAV binding in current study. Third, IFN-γ can also affect other components that are related to the structure of cell membrane, including cholesterol homeostasis (Zhou et al., 2020) and transmembrane proteins such as occludin, JAM-A, and claudin-1 (Utech et al., 2005). Depleting cholesterol with methyl-β-cyclodextrin (MCD) reduces influenza infection (Eierhoff et al., 2010). As jasplakinolide has no effect on cholesterol, disorganized membrane cholesterol may have contributed to the partial reversal of IAV binding and infection.

Our study reveals a mechanism of IFN-associated antiviral activity. The increasing knowledge on the IFN family has now demonstrated a wide range of overlapping, or complementary antiviral mechanisms from different IFNs to combat virus evasion (Hale et al., 2010; Garcia-Sastre, 2017). Further understanding of HA-glycan interactions and how the immune system (e.g. IFN-γ) regulates their interactions will advance the design of IAV binding/entry inhibitor.

### Limitations of the study

There are limitations in this study. First, in addition to actin, IFN-γ may cause reprogramming of cholesterol (Zhou et al., 2020), which may influence sialic acid cluster size and arrangement. Second, in addition to sialic acid, EGFR signaling also play a role in IAV entry (Eierhoff et al., 2010); therefore, the correlation of sialic acid cluster size, EGFR distribution, and its signaling on IAV binding requires further investigation.

## STAR★Methods

### Key resources table

**Table:** 

REAGENT or RESOURCE	SOURCE	IDENTIFIER
**Antibodies**		

Anti-influenza A antibody, nucleoprotein, clone A1	Millipore, Darmstadt, Germany	CAT#MAB8257; RRID: AB_95231
Influenza A H1N1 (Swine Flu 2009) Hemagglutinin/ HA antibody, rabbit Mab	Sino Biological, Wayne, USA	Cat#11055; RRID:AB_1960261
Goat anti-mouse IgG (H+L) Alexa Fluor 633	Thermofisher Scientific, MA, United States	Cat#A21050; RRID:AB_2535718
PE Goat Donkey anti-rabbit IgG, clone Poly4064	Biolegend, San Diego USA	Cat#406421; RRID:AB_2563484
Alexa Fluor 488 mouse IgG1, clone MOPC-21	Biolegend, San Diego USA	Cat#400129; RRID:AB_400129
Alexa Fluor(R) 488 anti-human EGFR antibody	Biolegend, San Diego USA	Cat# 352908; RRID:AB_11126165
Influenza A H3N2 (A/Brisbane/10/2007) Hemagglutinin / HA Antibody, Rabbit MAb	Sino Biological, Wayne, USA	Cat# 11056-R006; RRID: AB_2860296
Rabbit IgG Isotype Control antibody	Thermofisher Scientific, MA, United States	Cat# 31235;RRID:AB_243593
Mouse IgG2a kappa Isotype Control (eBM2a)	Thermofisher Scientific, MA, USA	Cat# 14-4724-82; RRID: AB_470114

**Bacterial and virus strains**		

A/Hong Kong/415742/2009(H1)pdm09	In house	N/A
A/Hong Kong/417610/2018 (H3N2)	In house	N/A
A/Vietnam/1194/2004 (H5N1)	In house	N/A

**Chemicals, peptides, and recombinant proteins**		

Trypsin from bovine pancreas, TPCK Treated	Merck, Darmstadt, Germany	Cat# T1426
UltraPure™ Low Melting Point Agarose	Thermofisher Scientific, MA, USA	Cat# 16520050
Recombinant human interferon gamma 1-b (IMMUKIN)	Boehringer Ingelheim, Berkshire, UK	N/A
Biotin Conjugated *Maackia amurensis* Lectin -MAA	EY Lab, San Mateo, USA	Cat# BA-7801-5
Biotin Conjugated *Sambucus nigra* (Elderberry Bark) -SNA-I	EY Lab, San Mateo, USA	Cat# BA-6802-1
Dulbecco’s modified eagle’s medium nutrient mix F-12 (DMEM F-12)	Thermofisher Scientific, MA, USA	Cat# 11320082
Eagle’s Minimum Essential Medium (MEM)	Thermofisher Scientific, MA, USA	Cat#11095080
Penicillin-Streptomycin (10,000 U/mL)	Thermofisher Scientific, MA, USA	Cat#15140122
HEPES	Thermofisher Scientific, MA, USA	Cat#15630080
PBS	Thermofisher Scientific, MA, USA	Cat#15630080
MEM (Temin's modification) (2X), no phenol red	Thermofisher Scientific, MA, USA	Cat#11935046
TritonTM X-100	Thermofisher Scientific, MA, USA	Cat#11332481001
ProlongTM Diamond Antifade with DAPI	Thermofisher Scientific, MA, USA	Cat#P36962
UltraPure™ 0.5 M EDTA, pH 8.0	Thermofisher Scientific, MA, USA	Cat#15575020
Zombie Violet^TM^ Fixable Viability Kit	Biolegend, San Diego, United States	Cat#423114
Alexa Fluor^TM^ 488 Phalloidin	Thermofisher Scientific, MA, USA	Cat#A12379
Propidium Iodide	Thermofisher Scientific, MA, USA	Cat#P3566
0.05% Trypsin EDTA	Thermofisher Scientific, MA, USA	Cat#25300-054
APC Streptavidin	Biolegend, San Diego USA	Cat# 405207
Streptavidin, Alexa Fluor™ 647 conjugate	Thermofisher Scientific, MA, USA	Cat# S32357
Jasplakinolide	Thermofisher Scientific, MA, USA	Cat#J7473
DMSO	Sigma	Cat#D2650
SYBR Premix Ex Taq (Tli RNaseH Plus)	Takara	CAT# RR420A
Crystal Violet	Sigma	CAT#C0775
Fetal bovine serum	ThermoFisher Scientific	Cat# 16140071
Bovine serum albumin Fraction V	Roche	Cat# 03116964001
Pierce™ 16% Formaldehyde (w/v), methanol free	ThermoFisher Scientific	Cat# 28908
Glucose	Sigma	G8270
Tris-HCL	Sigma	T3038
NaCl	Sigma	S9888
Cyclooctatetrane	Sigma	138924
beta-mercaptoethanol	Sigma	M6250
Glucose oxidase	Sigma	G0543
Catalase from *Aspergillus niger*	Sigma	C3515

**Critical commercial assays**		

QIAamp Viral RNA Mini Kit	Qiagen	CAT#52906
RNeasy Mini Kit	Qiagen	CAT#74106
D3 Ultra 8 DFA respiratory virus screening and identification kit	Quidel	N/A
CyQUANT™ MTT Cell Viability Assay	Thermofisher Scientific, MA, USA	Cat#V13154
AgPath-ID one-step RT-PCR	Life Technologies Limited	CAT#4387424

**Experimental models: Cell lines**		

A549	CCL-185	N/A
Calu3	HTB-55	N/A
MDCK	CCL-34	N/A

**Oligonucleotides**		

TNF Forward 5' CAAGGACAGCAGAG GACCAG 3'	Integrated DNA Technologies	N/A
TNF Reverse 5'TGGCGTCTGAGGG TTGTTTT3'	Integrated DNA Technologies	N/A
CXCL10 Forward 5'AGCAGAGGAA CCTCCAGTCT 3'	Integrated DNA Technologies	N/A
CXCL10 Reverse 5'ATGCAGGTACAG CGTACAGT 3'	Integrated DNA Technologies	N/A
IL-10 Forward 5' AACTGAGACATCA GGGTGGC3'	Integrated DNA Technologies	N/A
IL-10 Reverse 5'AAGGTTTCTCAAGG GGCTGG 3'	Integrated DNA Technologies	N/A
IL-6 Forward 5' GGCTGCAGGACA TGACAACT 3'	Integrated DNA Technologies	N/A
IL-6 Reverse 5' ATCTGAGGTGCC CATGCTAC 3'	Integrated DNA Technologies	N/A
GAPDH Forward 5' ATTCCACCCATGGCA AATTC 3'	Integrated DNA Technologies	N/A
GAPDH Reverse 5'CGCTCCTGGAAGAT GGTGAT3'	Integrated DNA Technologies	N/A
CCL2 Forward 5' GCTCATAGCAGCCAC CTTCATTC3'	Integrated DNA Technologies	N/A
CCL2 Reverse 5'GGACACTTGCTGCTG GTGATTC 3'	Integrated DNA Technologies	N/A
IFNB1 Forward 5'GCCGCATTGACCATCT 3'	Integrated DNA Technologies	N/A
IFNB1 Reverse 5' CACAGTGACTGTACT CCT 3'	Integrated DNA Technologies	N/A
IFNL1 Forward 5' CGCCTTGGAAGAGTC ACTCA 3'	Integrated DNA Technologies	N/A
IFNL1 Reverse 5' GAAGCCTCAGGTCC CAATTC 3'	Integrated DNA Technologies	N/A
IFNL2/3 Forward 5'AGTTCCGGGCCTG TATCCAG3'	Integrated DNA Technologies	N/A
IFNL2/3 5'GAGCCGGTACAGCCAATGGT3'	Integrated DNA Technologies	N/A
InfA-Forward 5’- GACCRATCCTGTCAC CTCTGAC -3’	Integrated DNA Technologies	N/A
InfA-Reverse 5’- AGGGCATTYTGGACAAA KCGTCTA -3	Integrated DNA Technologies	N/A
InfA Probe5’ FAM- TGCAGTCCTCGCTC ACTGGGCACG -BHQ1 3’	Integrated DNA Technologies	N/A

**Software and algorithms**		

Zen digital imaging for light microscopy	Zeiss	http://www.zeiss.com/microscopy/en_us/products/microscope-software/zen.html#introduction, RRID:SCR_013672
Fiji	ImageJ	http://fiji.sc, SCR_002285
FlowJo_V10	BD	https://www.flowjo.com/solutions/flowjo, SCR_008520
GrapgPad Prism 9	GraphPad	http://www.graphpad.com/, SCR_00279

**Other**		

2-2.4 μm polystyrene beads	Spherotech, Lake Forest, USA	CAT# PP-20-10
circle coverslip, 13 mm diameter	Thermofisher Scientific, MA, United States	CAT# CBAD00130RA1
Coverslip 18 mm, 0.13mm	Marienfeld Superior, Lauda-Königshofen, Germany	CAT# 111580

### Resource availability

#### Lead contact

Further information and requests for resources and reagents should be directed to and will be fulfilled by the lead contact, Kelvin Kai-Wang To (kelvinto@hku.hk).

#### Materials availability

This study did not generate new unique reagents.

### Experimental model and subject details

All cells were obtained from American Type Culture Collection (ATCC, Rockville, MD) and tissue culture reagents were obtained from Gibco (Gibco, Grand Island, NY, United States). A549 cells, CCL-185 and Calu3, HTB-55 were maintained in completed Dulbecco’s modified eagle’s medium nutrient mix F-12 (DMEM F-12). MDCK cells, CCL-34 were maintained in completed Eagle’s Minimum Essential Medium (MEM). All completed mediums were supplimented with 10% fetal bovine serum (FBS), penicillin (100 U/mL), streptomycin (100 μg/mL) and 25 μM 4-(2-hydroxyethyl)-1-piperazineethanesulfonic acid (HEPES). All cell lines were cultured at 37°C, 5% carbon dioxide (CO_2_). Calu3 and A549 cells have been authenticated by short tandem repeat (STR) profiling (Pangenia Lifesciences Limited). All cell lines were tested negative for mycoplasma contamination (The Centre for PanorOmic Sciences of the LKS Faculty of Medicine, the University of Hong Kong).

### Method details

#### 3-(4,5-Dimethylthiazol-2-yl)-2,5-Diphenyltetrazolium bromide (MTT) assay

3 × 10^4^ cells pre-treated with IFN-γ at indicated concentration at 37°C, 5% CO_2_. After 24 h, cells were washed twice with phosphate buffered saline (PBS) and resuspend cells in 90 μL of completed DMEM-F12. 10 μL of 12 mM MTT solution (Thermofisher Scientific, MA, USA) was added to each well. Incubate cells at 37°C, 5% CO_2_ for 4 h and added 100 μL of of SDS-HCL solution. Incubate cells for 18 h and read absorbance at OD570 nm.

#### IFN-γ, jasplakinolide treatment

For 24 h IFN-γ pre-treatment, 1 × 10^5^ A549 or Calu3 cells were seeded in 24 well plates in completed DMEM F-12 (Day 1) and cultured at 37°C, 5% CO_2_. After 24 h, completed DMEM F-12 medium was removed and cells were washed once with 1 mL PBS and replaced with serum free DMEM F-12 medium with or without Immukin, recombinant human interferon-gamma 1-b (IFN-γ1-b) (Boehringer Ingelheim, Berkshire, UK) at indicated concentrations (Day 2) to culture at 37°C, 5% CO_2_. After 24 h, cells were ready for experiment (Day 3). Immukin (IFN-γ1-b) used is referred as IFN-γ. For experiment with jasplakinolide treatment, IFN-γ was removed and washed twice with PBS. Jasplakinolide at indicated concentrations were added in 300 μL in plain DMEM-F12. Cells were incubated for 30 min (mins) at 37°C, 5% CO_2_ and followed by two washes with PBS.

#### Viruses propagation and cell infection

The IAV strains used in current study included A/Hong Kong/415742/2009(H1N1) (Yuan et al., 2019), A/Hong Kong/417610/2018 (H3N2) (Chen et al., 2019), A/Vietnam/1194/2004 (H5N1) (Zhao et al., 2018). IAV were propagated in MDCK cells in serum free MEM containing penicillin (100 U/mL), streptomycin (100 μg/mL) and 25 μM HEPES. For H1N1 and H3N2, culture medium was supplemented with 1 μg/mL trypsin from bovine pancreas, TPCK treated (Merck, Darmstadt, Germany). Infectious culture supernatant was collected after 24–36 h and titer was determined by plaque assay. For cell infection, cells were infected at indicated MOI in 200 μL of of virus. Cells were incubated at 37°C, 5% CO_2_ for 1 h. After the inoculum was removed, the cells were washed with 1 mL PBS once and replaced in serum free culture medium. Culture supernatant was collected after 24 h.

#### Plaque assay

Plaque assays were performed to determine titers of the virus stocks and infectious culture supernatant samples (Zhou et al., 2018; Chu et al., 2021). 1 × 10^5^ MDCK cells were seeded in 24-well plates. Confluent monolayers were inoculated with 175 μL of 10-fold serial dilutions of virus samples and incubated for 1 h at 37°C, 5% CO_2_. After the inoculum was removed, the cells were washed with PBS three times and overlaid with 1% ultraPure™ Low Melting Point Agarose (Thermofisher Scientific, MA, USA) in MEM, penicillin (100 U/mL), streptomycin (100 μg/mL). For H1N1 and H3N2, medium was supplemented with MEM and 1 μg/mL TPCK-treated trypsin (Merck, Darmstadt, Germany) and further incubated at 37°C, 5% CO_2_ for 72 h. The monolayers were fixed with 10% formaldehyde overnight at room temperature (RT). All fixed samples were then stained with 0.5% crystal violet in 25% ethanol/distilled water for 10 min for plaque visualization. Virus titers were calculated as plaque-forming units (PFU) per mL.

#### Time of addition study

A549 cells were infected with A/Hong Kong/415742/2009(H1N1) at MOI of  0.01 under three IFN-γ conditions: (i) Pre-exposure to cells; A549 cells were pre-treated with 0, 25 international unit (IU)/mL IFN-γ for 1 or 24 h (ii) Virus adsorption; 0, 25 IU/mL of IFN-γ were added to A549 cells at 0 h together with the viruses. For conditions (i) and (ii), cells were infected and incubated at 37°C, 5% CO_2_. After 1 h of infection, viruses were removed, cells were washed once with serum free DMEM F-12 and 0.5 mL of serum free DMEM F-12, 0.25 μg/mL TPCK-treated trypsin were added. (iii) Virus replication; cells were infected with viruses at 37°C, 5% CO_2._ After 1 h, viruses were removed, cells were washed once with serum free DMEM F-12 and 0.5 mL of serum free DMEM F-12 medium, 0.25 μg/mL TPCK-treated trypsin with or without 25 IU/mL of IFN-γ were added. For all three conditions, culture supernatant was collected at 24 h.p.i, and the antiviral activity was determined by plaque assay.

#### Cell-cell viral transmission assay

1 × 10^4^ A549 cells were first infected with A/Hong Kong/415742/2009(H1N1) at indicated MOI for 1 h at 37°C, 5% CO_2_. After 1 h, infected cells were washed with 1 mL PBS twice and 0, 100, 400 IU/mL IFN-γ pre-treated, un-infected A549 (5 × 10^4^) were added. Cells were co-cultured at 37°C, 5% CO_2._ After 24 h, cells were washed with 1 mL PBS for three times and fixed with 200 μL of 2% formaldehyde (Thermofisher Scientific, MA, United States) at RT for 1 h with orbital shaking. Cells were then permeabilized with 200 μL 0.1% Triton™ X-100 (Merck, Darmstadt, Germany). After 15 min of incubation at RT with orbital shaking, cells were blocked with 5% bovine serum albumin (BSA) in PBS for 1 h at RT. After blocking, cells were stained with 200 μL of 5 μg/mL anti-influenza A antibody, nucleoprotein, clone A1 or mouse IgG2a isotype control (Thermofisher Scientific, MA, United States) in 1% BSA in PBS for 1 h, with orbital shaking. Cells were then washed three times with 1 mL PBS. After wash, 200 μL of 4 μg/mL of goat anti-mouse IgG-AF633 and 0.3 μM Alexa Fluor™ 488 Phalloidin in 1% BSA in PBS were added to the cells for 45 min incubation at RT, with orbital shaking. Cells were washed three times with 1 mL PBS and mounted with Prolong™ Diamond Antifade with DAPI (Thermofisher Scientific, MA, United States). Samples were incubated in the dark at RT for 24 h before confocal analysis with Carl Zeiss LSM 880 (Zeiss, Jena, Germany). Confocal images were analysed with ZEISS ZEN Microscope software, Blue Edition (Zeiss, Jena, Germany).

#### Immunofluorescence assay

Immunofluorescence assay for viral protein expression was performed as instructed by the manufacturer. A549 cells were transferred into Teflon printed diagnostic slide and air-dried. Cells were then fixed in chilled acetone at −20°C for 10 min and stained with influenza A DFA Reagent, D3® Ultra 8TM DFA Respiratory Virus Screening and Identification Kit (Diagnostic Hybrids, Inc. Quidel, United States) following the manufacturer’s instructions and examined under epifluorescent illumination of an Eurostar III plus fluorescence microscope (EUROIMMUN AG, Lübeck, Germany).

#### Receptor analysis by fluorescene-activated ell sorting (FACS)

Unless stated, otherwise all reagents used were ice cold and samples were kept on ice at all time. 1 × 10^6^ A549 or Calu3 cells were blocked with 5% BSA, PBS on ice. After 30 min, cells were stained with either 50 μg/mL biotin conjugated *Sambucus nigra* (Elderberry Bark) -SNA-I (EY Lab, San Mateo, USA), or 50 μg/mL biotin conjugated *Maackia amurensis* Lectin –MAA (EY Lab, San Mateo, USA) or 0.25 μg of Alexa Fluor 488 mouse anti-human EGFR antibody, clone AY13 (Biolegend, San Diego United States), or 0.25 μg Alexa Fluor 488 mouse Ig gamma −1 (IgG1), clone MOPC-21 (Biolegend, San Diego United States) in 50 μL ice cold 1% BSA in PBS. Cells were incubated on ice for 1 h and washed three times with 2% BSA in PBS. After washing, 4 μg/mL allophycocyanin (APC) streptavidin (Biolegend, San Diego United States) were added to sample stained with biotin-MAA and biotin-SNA I. After 30 min of staining, cells were washed twice with 2% BSA in PBS and resuspended in 400 μL of 2% BSA in PBS containing 0.3 μg/mL propidium iodide (PI) for flow cytometry analysis. All samples were analysed with BD LSRFortessa^TM^ Flow Cytometer (Becton, Dickinson and Company, Frankin Lakes, United States). All data were analzed with Flowjo software, version 10 (Becton, Dickinson and Company, Frankin Lakes, United States).

#### Sialic acids and F-actin analysis confocal imaging

5 × 10^4^ A549 cells were seeded on 13 mm diameter sterilized circle coverslip (Thermofisher Scientific, MA, United States) and placed in 24 well plate with completed DMEM F-12. After 24 h of IFN-γ pre-treatment at 37°C, 5% CO_2_, the cells were washed with 1 mL ice cold PBS and blocked with 0.5 mL of 5% BSA in PBS on ice with orbital shaking. After 1 h, A549 cells were stained with 50 μg/mL biotin conjugated SNA I, or biotin conjugated MAA in 200 μL of ice cold 1% BSA in PBS on ice with orbital shaking. After 1 h, cells were washed three times with 1 mL of 1% BSA in PBS and stained with 200 μL of 4 μg/mL APC Streptavidin (Biolegend, San Diego United States) on ice with orbital shaking. After 30 min, cells were washed three times with 1 mL PBS and fixed with 200 μL of 2% formaldehyde (Thermofisher Scientific, MA, United States) at RT. After 1 h, cells were permeabilized with 200 μL of 0.1% Triton™ X-100 (Merck, Darmstadt, Germany) for 15 min at RT. Cells were washed once with 1 mL PBS and blocked with 200 μL of 5% BSA in PBS at RT with orbital shaking. After 1 h, cells were stained with 200 μL of 0.3 μM Alexa Fluor™ 488 Phalloidin (Thermofisher, Scientific, MA, United States) in 1% BSA in PBS at RT with orbital shaking. After 60 min, cells were washed three times with 1 mL PBS and mounted with Prolong™ Diamond Antifade with DAPI. Samples were incubated in the dark at RT for 24 h prior confocal analysis with Carl Zeiss LSM 710. Confocal images were analysed with ZEISS ZEN Microscope software, Blue Edition.

#### Virus attachment assay (FACS)

Unless stated, otherwise all reagents were ice cold and samples were kept on ice at all time. 2 × 10^5^ cells were washed with 1 mL of PBS once and pre-chilled on ice for 15 min. After chilling, cells were inoculated with H1N1, H3N2, H5N1 (MOI = 5) in plain DMEM F-12 and incubated on ice. After 1 h, cells infected with H5N1 were washed three times with PBS and lysed with RLT buffer for RT-qPCR for M gene detection. With cells infected with H1N1 and H3N2, cells were washed three times with 1 mL 2% BSA and blocked with 2% BSA in PBS on ice for 1 h. Cells were then stained with 1 μL of Zombie Violet (Biolegend, San Diego, United States) in 50 μL of 1% BSA in PBS. After 30 min of incubation on ice, cells were washed with 1 mL 2% BSA in PBS three times and stained with 1 μg of Influenza A H1N1 (Swine Flu, 2009) HA antibody, rabbit monoclonal antibody (SinoBiological, Wayne, United States), or 0.5 μg Influenza A H3N2 (A/Brisbane/10/2007) Hemagglutinin/HA Antibody, rabbit monoclonal antibody or rabbit IgG isotype control in 50 μL of 2% BSA, PBS and incubated on ice for 1 h. Cells were washed with 2% BSA in PBS three times and stained with 50 μL of 0.2 μg/mL goat anti-rabbit IgG-PE, clone Poly4064 (Biolegend, San Diego United States) in 2% BSA in PBS. Cells were incubated on ice for 1 h and followed by three washings with 1 mL PBS. Cells were fixed with 200 μL of 2% formaldehyde, PBS and incubated at RT. After 1 h of fixing, cells were washed once with 2% BSA in PBS, resuspended in 2% BSA in PBS and is ready for FACS analysis with BD LSRFortessa™ Flow Cytometer. Data were analysed with Flowjo software, version 10.

#### Virus attachment assay (confocal imaging)

5 × 10^4^ A549 cells were seeded on steriled 13 mm diameter circle coverslip (Thermofisher Scientific, MA, United States), and placed in 24 well plate with completed DMEM F-12, incubated at 37°C, 5% CO_2_. After 24 h of IFN-γ pre-treatment, cells were washed once with 1 mL of ice cold PBS and pre-chilled on ice for 15 min. After cell chilling, cells were inoculated with A/Hong Kong/415742/2009(H1N1) (MOI 50) in 200 μL ice cold plain DMEM F-12 and kept on ice with gentle orbital shaking. After 1 h of virus inoculation, cells were washed with 1 mL PBS three times and fixed with 200 μL of 2% formaldehyde for 1 h at RT, with orbital shaking. Cells were then permeabilized with 200 μL 0.1% Triton™ X-100 at RT for 15 min, with orbital shaking. Cells were washed once with 1 mL PBS and blocked with 300 μL 2% BSA in PBS for 60 min at RT, with orbital shaking. Cells were stained with 200 μL 5 μg/mL anti-influenza A antibody, nucleoprotein, clone A1 or mouse IgG2a isotype control (Thermofisher Scientific, MA, United States) in 1% BSA in PBS. After 1 h of incubation with orbital shaking, cells were washed three times with 1 mL PBS and stained with 200 μL of 4 μg/mL of goat anti-mouse IgG AF633 in 1% BSA in PBS. Cells were incubated for 45 min at RT, with orbital shaking. After washings three times with 1 mL PBS, cells were stained with 200 μL of 0.3 μM Alexa Fluor™ 488 Phalloidin in 1% BSA in PBS for 60 min at RT, with orbital shaking. Cells were then washed three times with 1 mL of PBS and mounted with Prolong™ Diamond Antifade with DAPI. Samples were incubated in the dark at RT for 24 h and samples were ready for confocal analysis with Carl Zeiss LSM 710. Confocal images were analysed with ZEISS ZEN Microscope software, Blue Edition.

#### dSTORM; pre-coating coverslide with polystyrene beads for drift control

Coverslip with thickness of 0.13 mm, circular diameter of 18 mm (Marienfeld Superior, Lauda-Königshofen, Germany) were cleaned with 70% ethanol and then placed on Cimarec + ™ Hotplate (Thermofisher Scientific, MA, United States) pre-set at 120°C. 100 μL of 2.0–2.4 μm polystyrene beads (Spherotech, Lake Forest, United States) in 50% ethanol were loaded onto each coverslip. Coverslips were heated at 120°C for 10 min and transferred to a storage dishes for experiment.

#### dSTORM; cell sample preparation

Coverslips pre-coated with polystyrene beads were sterilzed by exposing under UV light for 30 min in a biosafety cabinet. After UV sterilization, coverslips were placed in 12 well and 5 × 10^4^ cells were cultured on the coverslip in completed DMEM/F12.

#### dSTORM; sample staining

Cells were washed once with 2 mL 1% BSA in PBS and fixed with 300 μL of 4% formaldehyde at RT for 40 min (Chen et al., 2015). After fixing, cells were washed once with 1 mL, 1% BSA in PBS and then blocked with 5% BSA in PBS for 60 min. Cells were washed once with 1 mL, 1% BSA in PBS and stained with 300 μL of 50 μg/mL biotin conjugated SNA I or biotin conjugated MAA in 1% BSA in PBS, followed by incubation at RT with orbital shaking. After 1 h, cells were washed three times with 2 mL of 1% BSA in PBS and stained with 300 μL of 4 μg/mL Streptavidin, Alexa Fluor™ 647 conjugate (Thermofisher Scientific, MA, United States) with orbital shaking. After 30 min, cells were washed three times with 2 mL PBS and fixed with 300 μL of 2% formaldehyde for 20 min.

#### dSTORM imaging

dSTORM imaging was performed on Nikon inverted Eclipse Ti-E with a 100 × 1.49 N.A TIRF lens (Nikon, Tokyo, Japan), an objective TIRF illumination. The samples were imaged in freshly prepared buffer containing 10% glucose (Sigma, St. Louis, United States), 50 mM Tris-HCL pH8.0 (Sigma, St. Louis, United States), 10 mM NaCl (Sigma, St. Louis, United States), 2 mM cyclooctatetraene (Sigma, St. Louis, United States), 143 mM beta-mercaptoethanol (Sigma, St. Louis, United States), 0.65 mg/mL glucose oxidase (Sigma, St. Louis, United States) and 0.04 mg/mL catalase from *Aspergillus niger* (Sigma, St. Louis, United States). The coverslip that containing the seeded cells was sealed onto a microscopic depression slide (Sail Brand, China) using nail polish. The images were captured with excitation of a 640 nm laser with using an excitation filter (ZET532/647x, Chroma), a dichroic mirror (T760LPXR-UF2, Chroma) and an emission filter set (FF01-692/40–25, 25 mm, Semrock, NY, United States). Camera model, iXon Ultra 897 EMCCD (Andor Technology, Belfast, UK). A time series of 10,000 or 20,000 frames per cell was recorded for α-2,3-linked sialic acid and α-2,6-linked sialic acid. During the acquisition time, focus lock for x-y drift was applied with polystyrene beads pre-coated on the coverslip.

#### RNA isolation and reverse transcription quantitative polymerase chain reaction (RT-qPCR)

Total RNA was extracted from cells using Qiagen RNeasy Mini Kit according to manufacturer's instructions (Qiagen, Hilden, Germany). 200 ng of total RNA was reversed transcribed using PrimeScript RT Master Mix (Perfect Real Time) (Takara, Mountain View, United States). Quantitative PCR analysis was performed using Sybr®Premix Ex Taq (Takara, Mountain View, United States) with equal amount of cDNA and 0.3 μM each of forward and reverse primers. Primer sequences are listed in STAR Methods and cycling profiles are as follow. Cycling condition for glyceraldehyde-3-phosphate dehydrogenase (*GAPDH*), *CXCL10*, *CCL2*; pre-incubation: 95°C 30 s, amplification: 95°C 5 s, 60°C 30 s (45 cycles), melting: 95°C 5 s, 60°C 60 s, 95°C 1 s, cooling: 50°C 30 s. For *TNF*, *IL-6*, *IFNB1*, *IFNL1*, *IFNL2/3*; pre-incubation: 95°C 30 s, amplification: 95°C 5 s, 60°C 30 s, 82°C 2 s (40 cycles), melting: 95°C 5 s, 60°C 60 s, 95°C 1 s, cooling: 50°C 30 s. Samples were analysed with LightCycler®96 system (Roche, Basel, Switzerland). The present of the correct amplicons was verified by melt curve analysis. The cycle threshold were determined, normalized to levels of a constitutive housekeeping gene; GAPDH and used to obtain the relative levels of genes of interest using the 2^-ΔCt^ method.

#### Viral RNA isolation and qRT-PCR for M gene

140 μL of culture supernatant was collected and viral RNA was extracted using QIAamp Viral RNA Mini Kit (Qiagen, Hilden, Germany) according to the manufacturer's instructions. RT-qPCR was performed with AgPath-ID one-step RT-PCR (Thermofisher Scientific, MA, United States) according to the manufacturer's instructions with the InfA primers designed for M gene. Cycling profile is as follow; 50°C for 30 min, 1 cycle; 95°C for 2 min; 95°C for 15 s and 55°C for 30 s, 50 cycles. Samples were analysed with LightCycler®96 system (Roche). Absolute copy number was calculated from standard curves.

### Quantification and statistical analysis

All data are representative or mean of 2-4 independent experiments. Statistical significance was determined using unpaired T tests or multiple T tests in GraphPad Prism 9 software. ns – not significant, ∗p ≤ 0.05, ∗∗p ≤ 0.01, ∗∗∗p ≤ 0.001, ∗∗∗∗p ≤ 0.0001. p < 0.05 was considered statistically significant. Data are represented as mean ± SEM Error bar indicates SEM.

## Data Availability

All data reported in this paper will be shared by the lead contact upon request. This paper does not report original code Any additional information required to reanalyze the data reported in this paper is available from the lead contact upon request.
