# Supplementary material for: Interferon-gamma inhibits influenza A virus cellular attachment by reducing sialic acid cluster size
Source: iScience. 2022 Mar 6;25(4):104037. doi: 10.1016/j.isci.2022.104037 (PMC8938289; doi:10.1016/j.isci.2022.104037)
Supplement: Document S1. Figure S1 [file mmc1.pdf]

iScience, Volume *25*

Supplemental information

Interferon-gamma inhibits influenza A virus cellular attachment by reducing sialic acid cluster size

Carol Ho-Yan Fong, Lu Lu, Lin-Lei Chen, Man-Lung Yeung, Anna Jinxia Zhang, Hanjun Zhao, Kwok-Yung Yuen, and Kelvin Kai-Wang To

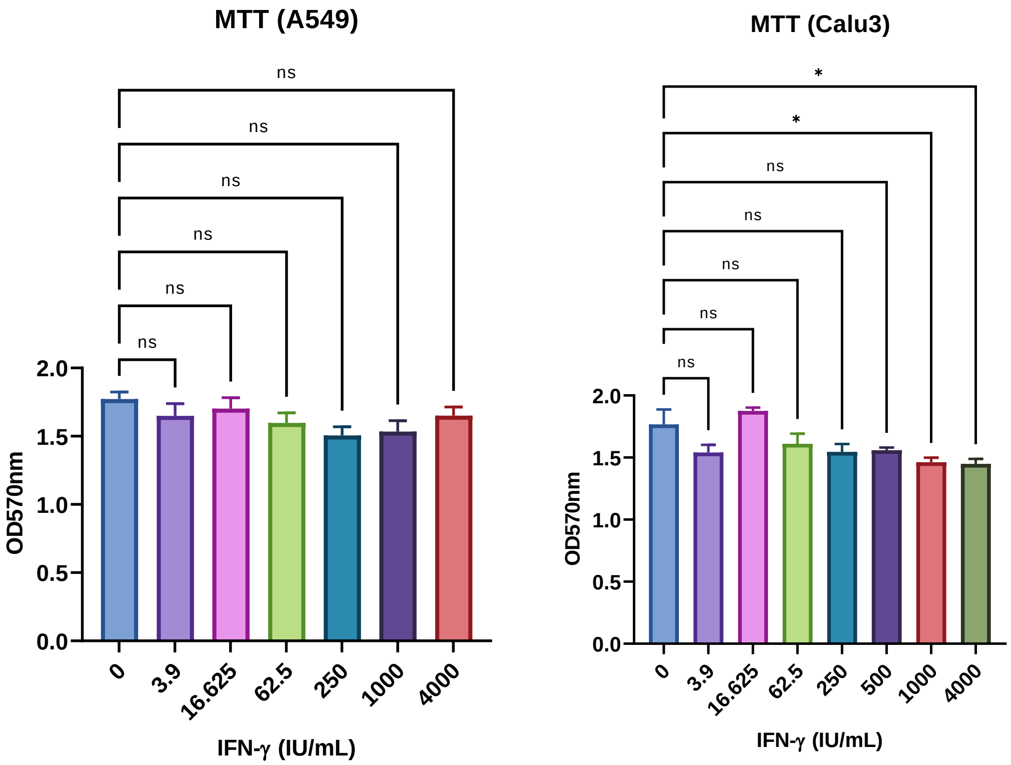

Figure S1: The viability of Calu3 and A549 cells after IFN-γ treatment. Related to STAR Methods and all figures.

Calu3 and A549 cells were pre-treated with IFN-γ for 24 h at indicated concentration and cell viability was tested by MTT assay. The optical density was analysed with wavelength 570 nm. The statistical significance from the average of 3 biological replicates is shown. Multiple T test was used to test statistical significance. ns not significant, * P≤0.05. Data are represented as mean +/- SEM. Error bar indicates SEM.
